# The Transcriptional Landscape of the Photosynthetic Model Cyanobacterium *Synechocystis* sp. PCC6803

**DOI:** 10.1038/srep22168

**Published:** 2016-02-29

**Authors:** Miguel A. Hernández-Prieto, Trudi Ann Semeniuk, Joaquín Giner-Lamia, Matthias E. Futschik

**Affiliations:** 1 Systems Biology and Bioinformatics Laboratory, Centre of Marine Sciences, University of Algarve, 8005-139 Faro, Portugal

## Abstract

Cyanobacteria exhibit a great capacity to adapt to different environmental conditions through changes in gene expression. Although this plasticity has been extensively studied in the model cyanobacterium *Synechocystis* sp. PCC 6803, a detailed analysis of the coordinated transcriptional adaption across varying conditions is lacking. Here, we report a meta-analysis of 756 individual microarray measurements conducted in 37 independent studies-the most comprehensive study of the *Synechocystis* transcriptome to date. Using stringent statistical evaluation, we characterized the coordinated adaptation of *Synechocystis*’ gene expression on systems level. Evaluation of the data revealed that the photosynthetic apparatus is subjected to greater changes in expression than other cellular components. Nevertheless, network analyses indicated a significant degree of transcriptional coordination of photosynthesis and various metabolic processes, and revealed the tight co-regulation of components of photosystems I, II and phycobilisomes. Detailed inspection of the integrated data led to the discovery a variety of regulatory patterns and novel putative photosynthetic genes. Intriguingly, global clustering analyses suggested contrasting transcriptional response of metabolic and regulatory genes stress to conditions. The integrated *Synechocystis* transcriptome can be accessed and interactively analyzed via the CyanoEXpress website (http://cyanoexpress.sysbiolab.eu).

The ability to adapt to an ever-changing environment is fundamental to successful life forms. Photosynthetic organisms, in particular, need to adjust various processes to daily changes in light intensity and nutrient availability. Thus, the capacity for adaption is a prominent feature of cyanobacteria—one of the most ancient life forms, and the only prokaryotes capable of performing oxygenic photosynthesis^1^. Cyanobacteria have successfully conquered most environments on earth, showing an amazing resilience to extreme conditions. In addition to their ecological importance as primary producers, cyanobacteria have attracted considerable interest as a potential source of natural products with high commercial value, including biofuels^2^.

Central to the adaptation process in cyanobacteria are stimulus perception, signal transduction, and consequent regulation of gene expression. Much insight into the mechanisms of gene regulation in cyanobacteria has been gained through the study of *Synechocystis* sp. PCC 6803 (hereafter *Synechocystis*). *Synechocystis* was the first photosynthetic organism, for which the genome was completely sequenced^3^. The early availability of its genome together with other advantages, such as easy genetic manipulation, fast growth, and ability to grow photo-heterotrophically, made *Synechocystis* a popular photosynthetic model organism. The sequencing of its genome also enabled the development of comprehensive cDNA and oligonucleotide microarrays^4^. As a result, numerous genome-wide expression studies have been performed for a variety of environmental conditions. However, these studies, accessible in major public repositories^5^, only gave insight into how cells react to single or a small number of related perturbations.

Integration of multiple transcriptome datasets permits a deeper understanding of how the cell regulates and coordinates the expression of genes involved in different metabolic pathways, triggered by divergent conditions. Compared to analyses performed on individual datasets, meta-analyses provide various advantages because they have: i) increased statistical power to detect co-expression; ii) robust *in silico* validation of expression profiles across studies; and, most importantly, iii) a rational basis to generate new hypotheses and to guide future experiments^6^. The first meta-analysis of transcriptomic data for *Synechocystis* included 163 microarray datasets on different environmental and genetic perturbations, and identified a set of genes commonly regulated under most perturbations, referred to as the Core Transcriptional Response^7^. However, a more thorough meta-analysis of expression of functionally related genes, in particular those associated with the photosynthesis, had not been carried out.

To exploit the existing wealth of *Synechocystis* transcriptomic data for the study of regulation of photosynthetic genes, we collected and integrated 756 available microarray-based transcriptome profiles from three public repositories, as the basis for the most integrative transcriptional study to date for *Synechocystis*. We took special care in the curation and pre-processing of these microarray data to minimize potential artifacts that might arise from the use of different microarray platforms by different laboratories. The integrated dataset composed of 722 microarrays (after curation) was clustered and examined for co-expression patterns to determine the activation and suppression of biological processes under various conditions. The global clustering of these expression data revealed significant enrichment in four functional gene clusters along the environmental perturbations analyzed: (i) Photosynthesis and respiration; (ii) General metabolism; (iii) Regulatory functions, transport and binding proteins; and (iv) Hypothetical proteins. A more focused analysis of these integrated expression data provided an unprecedented view of how the photosynthetic apparatus adapts to environmental changes in this cyanobacterium, and how photosynthesis is co-regulated with other processes on a system level. In particular, functional categories linked with light-dependent photosynthetic reactions were examined in detail, under a variety of environmental perturbations. In addition, we inspected the co-expression patterns of components of light-dependent *versus* light-independent reactions of photosynthesis with the aim to evaluate their transcriptional regulation. Finally, we extended and enhanced CyanoEXpress (http://cyanoexpress.sysbiolab.eu)-a web-based resource for *Synechocystis* transcriptomic data^8^-to facilitate independent analysis of the integrated expression data.

## Results

### Compilation and integration of gene expression datasets for *Synechocystis*

**Figure 1:**
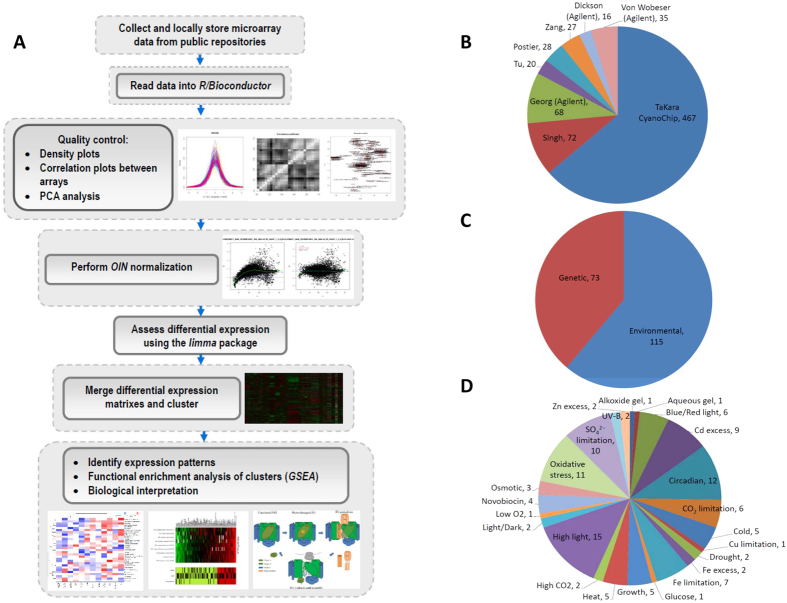
Data acquisition workflow and composition of integrated gene expression data used in this study. (**A**) Workflow of meta-analysis from data acquisition to biological interpretation. (**B**) Distribution of microarray measurements included in the integrated dataset derived from different platforms, (**C**) Number of expression contrasts obtained from WT (environmental) and mutant (genetic) *Synechocystis*, (**D**) Distribution of expression contrasts for different environmental conditions obtained from WT *Synechocystis*. WT = wild type.

The meta-analysis of microarray data generated by different research groups, using different platforms and under multiple environmental conditions requires a set of standardized steps to assure reliability of the derived results (Fig. 1A). The compiled public data consist of 756 microarray measurements carried out on seven different microarray platforms (Fig. 1B). The most commonly used platform was the CyanoCHIP from Takara Institute (467 microarrays), followed by those described by Georg (78), Singh (72), von Wobeser (35), Postier (28), Zang (27), Tu (20), and Dickson (16). The vast majority of microarray measurements (ca. 86%) cover genes encoded in the *Synechocystis* chromosome, neglecting genes in the seven mega plasmids. Altogether, probes against 3064 common genes were present within the different platforms (see Supplementary file 1 online for a more detailed description). We only included microarray experiments for which raw data were available; this requirement permitted us to establish a standardized procedure for quality assessment and normalization (Supplementary file 1; Fig. S1–S6). Measurements that displayed persistent artifacts were excluded (see Supplementary file 1 for details). In total, 722 of the initial 756 microarray measurements were retained for meta-analysis.

The collated microarray data can be broadly divided into two types of perturbations: environmental and genetic, depending on whether the experiments were carried out with Wild Type (WT) or genetically modified strains (Fig. 1C). Statistical evaluation of all collated experiments, in which transcriptional changes were measured after environmental perturbations, enabled us to define 115 different contrasts. These contrasts originated either from experiments with a single time point (19) or from time series experiments (15). To facilitate interpretation, environmental contrasts were categorized based on the type of perturbation (Fig. 1D). An additional 73 contrasts were defined by evaluating experiments, comparing the gene expression between deletion mutants and WT *Synechocystis* under different environmental conditions (Fig. 1C). Most of the included deletion mutants were defective in a member of their two-component systems, which play important roles in perception and transduction of environmental cues in prokaryotes^9^ or in Serine/Threonine kinases regulating functions such as cellular motility and salt acclimation^10^. Table S1 provides information on different mutants represented in the integrated dataset.

### Cluster analysis of meta-data set reveals the transcriptome landscape of *Synechocystis*

To study the coordinated regulation of gene expression due to different perturbations, we carried out hierarchical two-way clustering of the integrated expression data. To avoid effects related to the genomic background of the strain used in the studies, we excluded all experiments carried out using non-WT strains as these showed a distinct expression pattern (Supplementary Fig. S7). This observation corroborates previous reports that suggest disruption of the two-component system, involving either the sensor histidine kinase or the response regulator, can affect the expression of a large range of genes^11^,^12^, resulting in strong phenotypes. For readers interested on those genes, clustering of the full dataset can be explored using the CyanoEXpress website.

**Figure 2:**
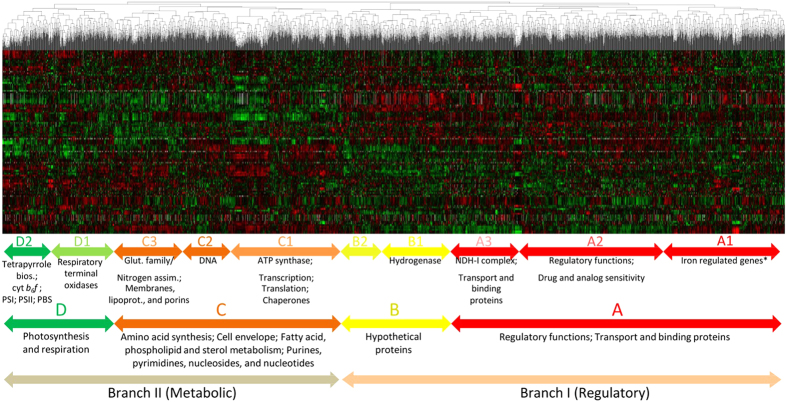
Global view of clustered differential gene expression data. Hierarchical clustering was applied to 3064 *Synechocystis* genes based on expression data obtained for perturbations in environmental conditions. The dendrogram at the top displays the structure of clusters produced, while the molecular functions enriched among the genes of these clusters are shown below. Positive or negative expression contrasts with the respect to the corresponding reference samples are indicted by shades of red or green, respectively.

To facilitate exploration of clusters, we represented the variations in gene expression as a heat map (Fig. 2), which can be explored through the CyanoEXpress website by selecting only environmental perturbations. The emergence of various large swatches of the same color indicated that numerous groups of genes shared similar expression patterns over multiple contrasts (or environmental perturbations). Inspection of their corresponding dendrogram revealed a separation of genes into two main branches. To investigate whether this separation reflected gene function, we examined the statistical enrichment of genes with common function within these different branches, using their functional classification in Cyanobase (Table S2). Indeed, we found that the main two branches (I and II) in the dendrogram were significantly enriched in genes encoding proteins assigned to the categories: (I) ‘Transport and binding proteins’ and ‘Regulatory functions’; and, (II) genes linked to metabolic categories (Table S3). Further division of both branches, hereafter “regulatory” and “metabolic” branches, revealed that genes belonging to the same functional sub-category tended to group together (Fig. 2).

The “regulatory” branch formed by 1715 genes is subdivided in two main clusters: A and B. Cluster A splits into three further sub-clusters: A1 (442 genes), A2 (850 genes), and A3 (423 genes). A1 is the only sub-cluster not statistically enriched in any specific functional category, although it contains genes that are part of the regulon of the Ferric uptake regulator (FurA), as described in previous work^13^. In contrast, sub-clusters A2 and A3 are both significantly enriched in several categories, i.e., ‘Regulatory functions’, ‘Transport and binding proteins’, and the subcategory ‘NDH-I complex’ (denoted ‘NADH dehydrogenase’ in Cyanobase). Meanwhile, cluster B contains a large number of hypothetical proteins, with a sub-cluster enriched in genes of the subcategory ‘Hydrogenase’.

The “metabolic” branch contains 1323 genes, divided into two main clusters: C and D. Cluster C with 890 genes is enriched in genes encoding enzymes involved in general cell metabolism, including transcription, translation, and amino acid synthesis. Finally, cluster D with 433 genes is significantly enriched in genes encoding subunits of the two photosystems (PSI, PSII), the cytochrome *b*_*6*  _*f* complex, the phycobilisomes (PBS), respiratory terminal oxidases, and enzymes involved in the synthesis of tetrapyrrole compounds that are prosthetic groups in these complexes. This cluster constitutes the most important cluster for the study of the regulation of photosynthesis as discussed in detail below. Overall, clustering showed that the integrated data provide a powerful basis to distinguish between a variety of gene expression profiles, and that genes encoding functionally related proteins tend to group together (despite the heterogeneous origin of the datasets).

### Coordinated adjustment of molecular processes under different environmental effects

**Figure 3:**
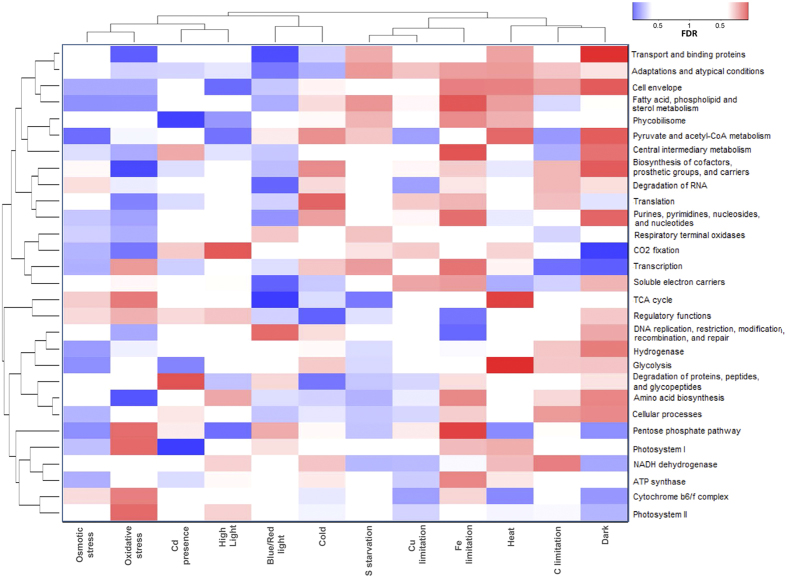
Graphical representation of the adaptive response of molecular processes to different environmental stimuli. The heat map shows activation (as shades of red) and repression (as shades of blue) for *Synechocystis* genes grouped within cellular functional categories under different types of environmental perturbations. The color intensities are defined by the calculated significance expressed as False Discovery Rates (FDR), as outlined in the Materials and Methods. Darker shades indicate a higher significance of transcriptional change. An equivalent graphical representation of the adaptive response, including all functional subcategories is provided in Supplementary Fig. S8.

Environmental perturbations lead to activation, modulation or repression of molecular processes. Although such behavior has been extensively studied for specific conditions, how cellular processes are orchestrated under a wider range of perturbations has not been systematically addressed in *Synechocystis*. To assess whether there is a coordinated transcriptional response to such perturbations, we grouped the expression contrasts into 12 different classes of environmental perturbations (Table S1): high-intensity light, red-blue light, dark, copper limitation, iron limitation, cadmium presence, carbon (C) limitation, sulfate starvation, heat, cold, oxidative stress (H_2_O_2_, DCMU, DBMIB, methyl viologen), and osmotic stress (NaCl, sorbitol). Subsequently, we calculated the significance of gene expression changes within the functional gene categories (Table S2) for each of these 12 classes. For this purpose, we ranked all contrasts, with respect to their average value within a functional category, and determined whether contrasts associated with a certain type of perturbation showed a tendency towards upper or lower ranks. To facilitate assessment of the impact of environmental perturbations on molecular processes, we performed a two-way clustering of functional categories and perturbations based on the calculated significance for activation or repression changes summarized in the Fig. 3. Inspection of the transcriptional response to these stresses at system-level revealed that osmotic, oxidative stress and blue/red light negatively affected gene expression in most categories. In contrast, heat, or iron limitation tended to increase transcription of most functional categories. An interesting effect was observed for dark conditions, where most functional categories were either highly repressed or induced in *Synechocystis*. This observation highlights how oscillation from photosynthetic to respiratory metabolism leads to a dramatic change in global expression, affecting almost all cellular processes.

Our results suggest that the impacts of different environmental perturbations differ in magnitude among functional categories. For example, environmental perturbations that severely affect membrane integrity, i.e., S or Fe limitation, and heat, result in the accumulation of genes encoding proteins functionally linked with the cell envelope, fatty acid and phospholipid synthesis, as well as transport and binding. Another notable adaptive pattern was observed for hydrogenase-related genes that were highly repressed under osmotic stress and induced under low C and dark conditions. A similar regulation has been previously reported for *Anabaena sp*. PCC 7120^14^, indicating conserved regulation of hydrogenase genes among different cyanobacteria groups. As expected, genes encoding enzymes associated with respiration tend to be up-regulated in cells grown in the dark, while genes encoding photosynthetic proteins are down-regulated. Transcription of genes within the functional category ‘respiratory and photosynthesis’ was induced under perturbations leading to increased levels of reactive oxygen species (Fe limitation, high light and oxidative stress; Fig. 3).

In general, genes encoding PSII subunits showed similar fluctuations to genes encoding subunits of the cytochrome *b*_*6  *_*f* complex, while adenosine triphosphate (ATP) synthase genes behaved similarly to genes encoding subunits of the NDH-1 complex. Intriguingly, the functional subcategory ‘CO_2_ fixation’ placed closer to the category ‘respiratory terminal oxidases’ than to the subcategories for photosynthetic complexes in the dendrogram. Their co-regulation might be explained by the fact that both processes function as electron sinks in the electron transport chain^15^.

### Differential regulation of photosynthetic genes upon environmental changes

In general, we observed that transcripts of functional sub-categories related to light harvesting, electron transfer, and conversion of light into chemical energy (via ATP and NAD(P)H) undergo significantly larger quantitative changes than other functions (Table 1). Similar magnitude changes were also observed for genes in sub-categories crucial to the response to stress conditions, including the sub-categories ‘Adaptation and atypical conditions’, ‘Chaperones’, ‘Transcription’ and ‘Translation’. In contrast, some functional categories such as ‘DNA replication, restriction, modification, recombination, and repair’; ‘Regulatory functions’, and ‘Metabolism of aromatic amino acids’ showed more constitutive expression, with expression changes that were significantly smaller than expected by chance (Table 1).

Given the importance of *Synechocystis* as a photosynthetic model organism, we investigated the regulation of photosynthetic sub-categories in response to environmental perturbations in more detail. Our approach identified environmental conditions that promoted expression trends in part or in all of the genes within selected functional sub-categories. To do this, we generated ranked expression matrices and represented their results as heat maps, using the perturbations as a barcode. Using these maps, we could identify coordinated regulation across genes within a functional category, as well as the environmental perturbations that lead to their uniform transcriptional changes.

**Figure 4:**
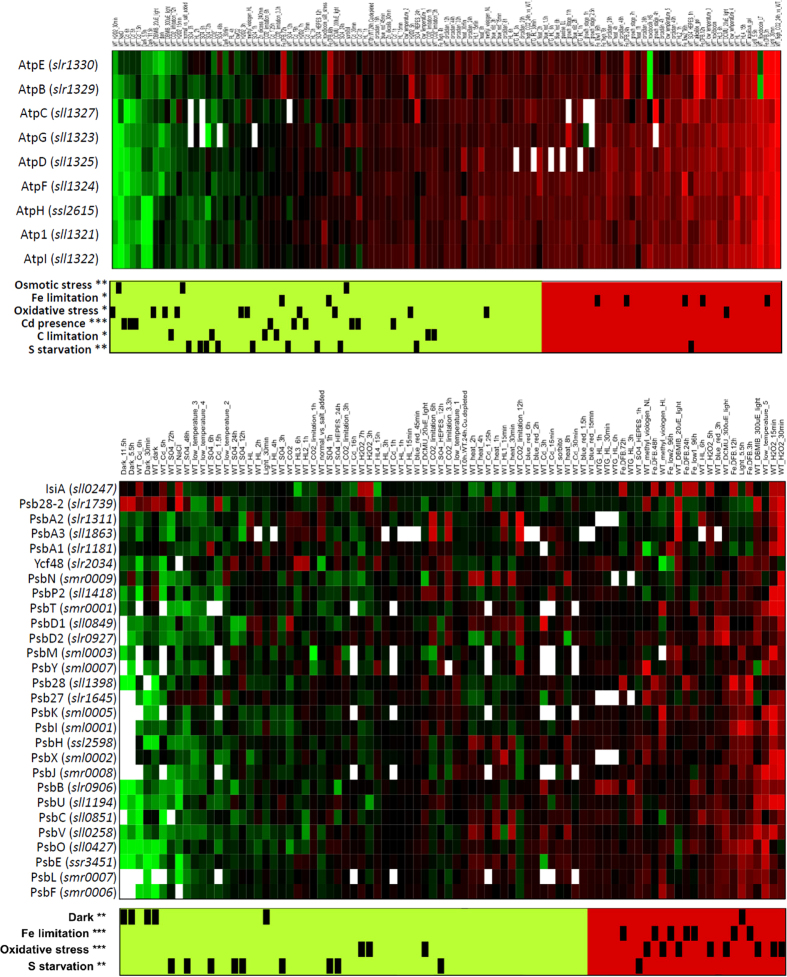
Functional subcategories corresponding to the adenosine triphosphate (ATP) synthase (top panel) and photosystem II (PSII) complexes (bottom panel). Contrasts are sorted such that their average value increases from left to right. Associations between contrasts and perturbation types are indicated in the barcode below the heat maps. Only perturbations for which the associated contrasts show significant deviation from a random distribution are displayed. The significance is indicated by stars: *** for p-value < 0.001, ** for p-value < 0.01, and *for p-value < 0.05.

Typically, functions performed by multimeric complexes had consistent expression changes across their associated genes, indicating a strong co-regulation. This was especially the case for genes encoding subunits of the ATP synthase and PSII (Fig. 4). The gene encoding subunits of the ATP synthase were significantly repressed upon addition of cadmium, as well as under osmotic stress and sulfate limitation, consistent with previous reports^16^,^17^ (Fig. 4A). Interestingly, under Fe limitation, expression of genes associated with ATP synthase tended to be up-regulated. In PSII, the genes encoding its subunits were up-regulated during Fe limitation or oxidative stress, and down-regulated in the dark or upon sulfate starvation. Nevertheless, two genes included in Cyanobase within the PSII subcategory had very distinct activations: the gene *sll0247* encoding the Fe stress induced (IsiA) protein, and *slr1739* encoding psb28-2. Both genes showed increased expression, whenever overall expression of PSII genes decreased (Fig. 4B).

**Figure 5:**
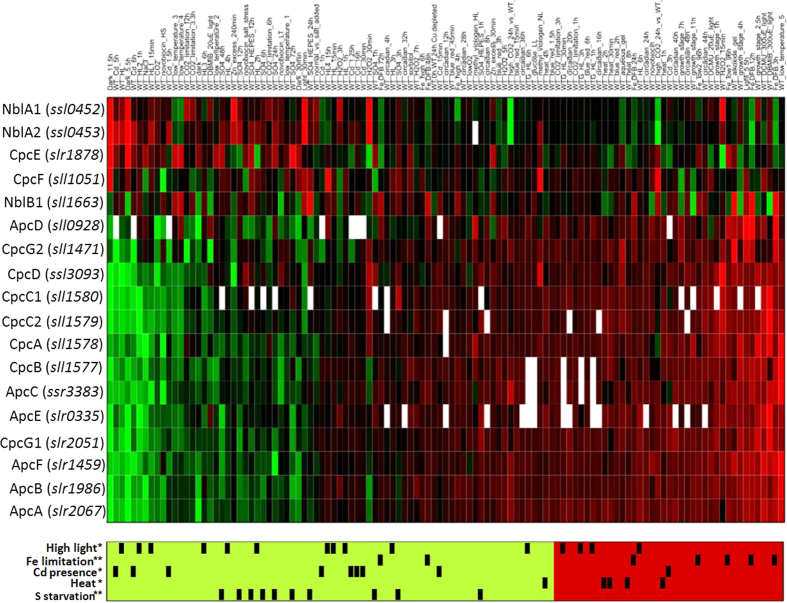
Heat map of the genes grouped under the functional subcategory ‘Phycobilisome’. Contrasts are sorted, such that their average value increases from left to right. Associations between contrasts and perturbation types are indicated in the barcode below the heat map. Only perturbations, for which the associated contrasts showed significant derivation from the distribution expected by chance, are displayed. Significance is indicated by stars: ** for p-value < 0.01, and * for p-value < 0.05.

In addition to the more typical homogenous expression profiles observed within functional sub-categories, we also detected categories that displayed heterogeneous profiles. In some cases, the reason for such heterogeneity was evident, especially when chaperones or proteases involved in the assembly or degradation of functional complexes were included in a subcategory. Likely, expression of these genes is transient and is not required, once the cell has successfully adapted to new conditions. This is illustrated by the functional subcategory PBS, which includes genes, *nblA1* and *nblA2*, encoding the subunits of a heterodimeric protease involved in its degradation, as well as *cpcE*, *cpcF* and *nblB1* that code for lyases linked with both the assembly of the chromophore into the apoprotein and its degradation^18^ (Fig. 5). These five genes were strongly induced by high-intensity light, the presence of cadmium or sulfur starvation, when genes coding structural components of the PBS were repressed. This observation suggests that an extra subdivision might be required to de-convolute co-regulation patterns. It also might explain why the subcategory PBS did not cluster together with the photosystem complexes, the cytochrome *b*_*6  *_*f* or the ATP synthase in Fig. 3. Readers interested in other functional categories and their transcriptional responses not included here could use the search tool in CyanoEXpress to query the expression of multiple genes at once.

### Predicting genes linked to photosynthesis through co-expression analysis

Despite being the most studied cyanobacterium, the embedding of photosynthesis in the complete cellular network to guarantee the cellular homeostasis in *Synechocystis* is not well understood. We expect that genes playing an important role in the coordination of photosynthesis with other cellular processes will show similarity in their expression patterns to photosynthetic genes indicating coordinated regulation. Thus, we identified genes whose expression is highly correlated with at least one of the eight functional sub-categories of ‘Photosynthesis and respiration’ as annotated in Cyanobase, but which were not associated with the same sub-category. Such genes might constitute nodal points connecting different processes in the cellular network of *Synechocystis*. Since the eight categories show different strength of internal co-expression, individual thresholds were set for association of genes with these categories. For instance, genes associated with the PBS tend to be tightly co-expressed, so a higher threshold was set for the ‘PBS’ sub-category than e.g. for genes associated with ‘CO_2_ fixation’, which tend to display weaker co-expression (see *Methods* section). In total, 401 connections of genes to sub-categories of ‘Photosynthesis and respiration’ (including those between sub-categories) were found (Supplementary Table S5). Notably, we identified 96 connections for PSI, PSII and PBS based on strong correlation. The corresponding genes might provide interesting starting points for future investigations, how the photosynthetic machinery is internally coordinated and linked to other processes.

## Discussion

**Figure 6:**
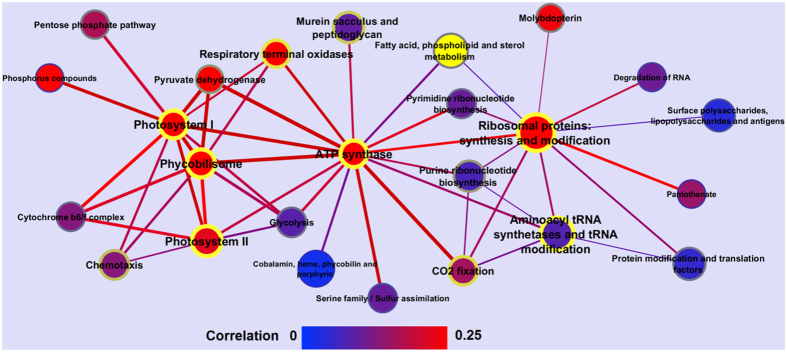
Network of molecular processes based on co-expression. Nodes represent molecular processes, while edges represent the calculated average Spearman correlation between the associated genes. The size of the nodes is proportional to the number of genes associated with a particular process. Node and edge colors represent the average correlation of genes within and between processes, with respect to a gradient color bar from blue to red. Font size and color of node border (blue to yellow) indicate the z-score for the average co-expression of genes within the same processes, while the widths of edges indicate the log odds of observing highly correlated gene pairs between two processes.

Given *Synechocystis’* importance to our understanding of photosynthesis and its potential for biotechnological applications, we sought to resolve its transcriptional landscape by evaluating gene expression across a diverse range of environmental conditions. In general, we found a highly coordinated transcriptional adaption of *Synechocystis* to environmental stress conditions, covering a variety of processes. To help visualize the level of co-transcription of the different functional categories, we created a network using the expression correlation between the genes within each category (Fig. 6). Notably, some processes seemed to play a more central role in the cell, having more interconnections with other functional categories. Indeed, our network shows PSI, PSII, PBS, ATP synthase, and the ribosomal proteins as central nodes, sharing regulatory mechanisms with components of various other functional categories. Intriguingly, these central functions are also among those with the highest variability in expression (Table 1; see also Table S4 for a more complete listing). Moreover, we see highly significant co-expression of photosynthesis, ATP synthase, and translation, demonstrating a tight coordination between the photosynthetic apparatus and protein synthesis (Fig. S8). This representation points to a dynamic core of essential functions that undergo extensive transcriptional reprogramming under stress conditions, but still serve as central connectors of the cellular network.

A more detailed view of the coordination of individual photosynthetic components can be found in the clustering of the 115 expression contrasts for environmental perturbations, which resolved the pool of transcripts into four main gene clusters (Fig. 2). Functional enrichment analyses of these clusters revealed a region (cluster D in Fig. 2), where genes associated with photosynthesis and respiration were significantly more abundant. Remarkably, the structure of this cluster closely reflected the known light dependency of reactions, in which these genes participate. More specifically, genes encoding subunits of complexes traditionally classified as part of the “light-dependent” photosynthetic reactions, PSI, PSII, PBS, and the cytochrome *b*_*6  *_*f* complex were grouped together, showing evident co-regulation under different environmental perturbations. In contrast, genes encoding the central enzymatic complex of the “light-independent” photosynthetic reactions and the ribulose-1,5-bisphosphate carboxylase/oxygenase (RuBisCo) complex formed a separate cluster, together with genes known to be up-regulated under CO_2_ limiting conditions (Supplementary Fig. S9). In the following discussion, we illustrate how gene clusters can be used to clarify the regulation of key photosynthetic genes, and to connect components and gene families associated with photosynthesis, based on their co-expression.

**Figure 7:**
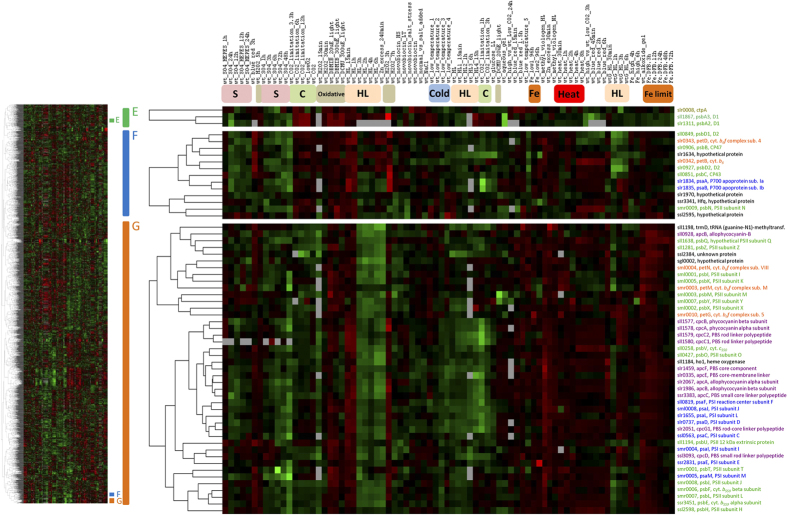
Clusters containing genes encoding photosystem II subunits. The clusters correspond to regions of the global cluster shown in Fig. 2 (a thumbnail image of the global cluster is shown on the left to localize the clusters discussed in the text). The gene names have been colored to facilitate the visualization of genes encoding proteins for different photosynthetic complexes, with PSII in green, PSI in blue, PBS in purple, Cyt. *b*_*6*_ *f* in orange, and other categories in black. The gene associations arose through comparison of all the samples using wild-type strains grown under different environmental perturbations. Cluster E containing *psba3* and *psba2* genes encoding the PSII reaction center D1 protein. Clusters F and G significantly enriched in genes encoding subunits of different photosynthetic complexes. Color bars positioned under the different contrasts indicate modification of the same environmental factor; S: Sulfate starvation, C: Carbon limitation, HL: High-intensity light, Oxidative: Oxidative stress, Cold: Low temperature, Fe: Iron limitation. Other conditions discussed in the text were trimmed for publication.

One of the most important factors determining the expression levels of photosynthetic genes appears to be oxidative stress. This is especially true for PSII, which has been shown to be the main source of reactive oxygen species, as well as the most frequent target of photodamage^19^. Our analysis shows that PSII-related genes are up-regulated under conditions of high oxidative stress, including high-intensity light and Fe limitation, but repressed under dark conditions (Fig. 3). The reason behind the increased transcription of genes encoding PSII subunits under oxidative stress seems to be the higher PSII repair rate under these conditions^19^,^20^,^21^. In fact, it has been frequently observed that genes encoding the D1 subunit of PSII accumulate under high-intensity light^22^,^23^. The *Synechocystis* genome contains gene duplications for two of the four PSII core subunits, with three gene copies for D1 (*psbA1*, *psbA2*, *psbA3*) and two for D2 (*psbD1*, *psbD2*). Interestingly, while it has been reported that the different *psbA* alleles are regulated differently^24^,^25^,^26^, inspection of the integrated expression data revealed an overall similarity in their transcriptional changes (Fig. 7). Nevertheless, under conditions where oxygen is not limiting, only D1 proteins encoded by *psbA2* or *psbA3* genes have been detected^24^,^27^. Analysis of single mutants lacking one of these two genes showed that they are able to replace each other, without obvious phenotypic differences^22^. Consistent with these observations, a high degree of expression correlation between *psbA2* and *psbA3*, compared to all other PSII subunits was observed (Fig. 7; cluster E). The independent clustering of *psbA2* and *psbA3* from other genes encoding structural PSII subunits (Fig. 7; cluster F and cluster G) is probably due to the high turnover of D1 under stress conditions compared with other subunits, and the capacity of the cell to substitute it at a monomer level^21^,^28^. It also indicates a regulatory mechanism at transcriptional level that is distinct from those for other PSII subunits.

We also detected a large cluster (part of cluster D in Fig. 2) containing 43 genes: 15 genes coding for PSII subunits, 8 genes coding for PSI subunits, 3 genes coding for cytochrome *b*_*6  *_*f*, 12 genes coding subunits of the PBS antenna, and 3 genes with no annotated function (*sll1638*, *ssl2384*, *sgl0002*) (Fig. 7; cluster G). Notably, this large cluster did not include the genes *sll0849* and *slr0927*, encoding the inner core PSII subunit D2 or the genes encoding the inner antennae CP47 (*slr0906*) and CP43 (*sll0851*). Instead, these genes clustered together with others, forming a separate group of 13 genes (Fig. 7; cluster F), along with *smr0009* encoding the PSII subunit PsbN, two genes encoding cytochrome *b*_*6*_*  f* subunits, two encoding the PSI core subunits PsaA and PsaB, and four encoding proteins with no annotated function (*slr1634*, *slr1970*, *ssr3341*, *ssl2595*).

The tight clustering of seven functionally unannotated genes (from clusters F and G) with known components of the photosynthetic apparatus suggests a potential functional association of these genes with photosynthesis. Indeed, bibliographic and database searches carried out for these unannotated genes support this hypothesis. For example, of the three unannotated genes in cluster G, *sll1638*, although not annotated in Cyanobase, encodes the PsbQ subunit of PSII^29^. The other two genes encode putative membrane-bound proteins, with two (*ssl2384*) and one (*sgl0002*) predicted hydrophobic domains, implying a possible location in the thylakoid membrane. Furthermore, *sgl0002* is located in the same orientation and genomic region as *sml0004* encoding the cytochrome *b*_*6  *_*f* subunit VIII^30^. Intriguingly, within the unannotated set in cluster F, we detected the operon formed by *slr1970* and *ssr3341*, with the latter encoding an Hfq homolog related to degradation of non-coding RNAs and cell motility^31^, but no reported previous links to photosynthesis (Fig. 7; cluster F). Of the other two unannotated genes in cluster F, *ssl2595* is encoded in adjacent position on the chromosome, but in opposed direction to *psbN*, which is located in the sister branch of the dendrogram, due to the high expression correlation between both genes. These examples indicate that the association of genes in clusters can be used to uncover novel genes associated with photosynthesis.

**Figure 8:**
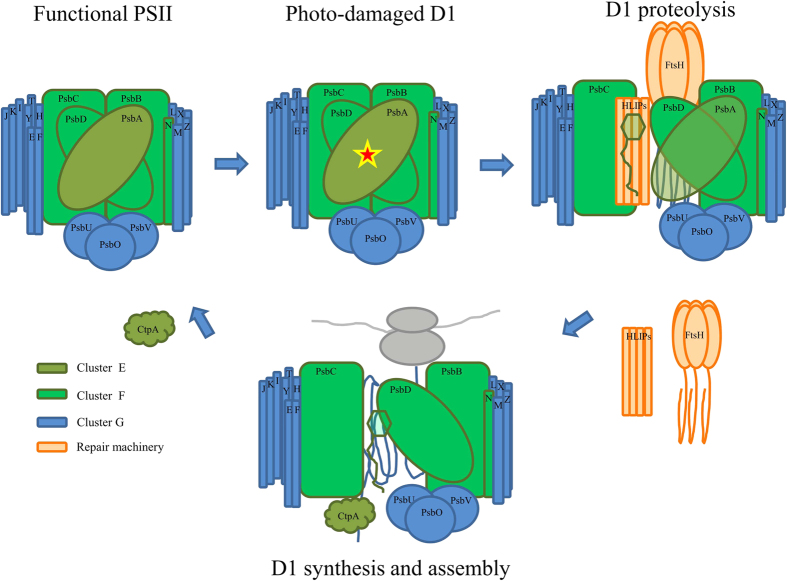
Potential model of the repair cycle of a photo-damaged D1 protein. The exposure of cells to stress conditions in the presence of light often results in irreversible oxidation of D1, which subsequently needs to be replaced. In the repair process, several proteases and chlorophyll-binding proteins are recruited at different stages, as previously suggested^39^,^40^. The observed co-expression suggests a cooperative role of FtsH proteases and high-light induced proteins (HLIPs) in the D1 proteolysis, as indicated in the scheme. The color of the different subunits and recruited proteins symbolizes their pertinence to differently regulated gene clusters.

The separation of various photosynthetic components into different clusters suggests different sets of regulatory factors for assembly and maintenance of PSI and PSII. We found members of two gene families (FtsH proteases and high-light induced proteins; HLIPs) placed in the same cluster. It is well known that FtsH proteases are required under stress conditions to repair or assemble subunits of damaged PSII. For example, mutants defective in FtsH2 (slr0228) exhibit an increased sensitivity to high-intensity light^32^ and UV-B light^33^, probably because of inefficient repair of photodamaged PSII. Both FtsH2 and FtsH3 (slr1604) have been co-purified by pull-down assays, with PSII complexes^32^,^34^. Recent evidence has shown FtsH3 to be an essential component of isolated FtsH hetero-oligomeric complexes, partnering not only FtsH2, but also FtsH1 (slr1390)^35^. Notably, after exposure to high light, HLIPs were co-isolated with PSII and FtsHs^36^. Co-purification of PSII with HLIP, FtsH2, FtsH3, and FtsH4 under native conditions indicated that HLIPs might perform a protective role during PSII biogenesis under stress^39^,^40^,^41^. If this is the case, and both gene families are required for an efficient assembly of PSII, then a co-transcriptional regulation can be expected. Indeed, we observed similar transcription profiles of genes encoding FtsH2, FtsH3, HliC (ssl1633), HliA (ssl2542), and HliB (ssr2595) under different environmental conditions (Fig. S10; cluster H). The cluster structure appears to correspond well with results from pull-down experiments involving tagged HLIPs under native conditions. Physical interaction between these proteins, together with the results of our hierarchical clustering, reinforces the idea of a complementary role for these two families during PSII assembly (Fig. 8).

Another indication provided by the clustering is the potential co-assembly of both photosystems. Co-regulation, inferred by the clustering of genes encoding PSI and PSII subunits (Figs 2 and 6), supports the idea that there is an intermediate assembly unit formed by PSII and PSI subunits^42^,^43^. Such a putative assembly unit would facilitate the quenching of absorbed solar energy during the assembly or repair of both complexes, mitigating photo-damage. This idea originates from pull-down experiments and native separation of Psb27 (slr1645) containing complexes, which co-purified CP43 bound to PSI subunits^44^. Our results also point to a functional connection, since the expression profile for *psb27* closely resembled that of *slr0171* (*ycf37*) (Fig. S10; cluster J), for which the corresponding protein contributes to assembly and stabilization of PSI complexes^45^,^46^. Likewise, the co-transcription of *psbN* with genes encoding core subunits of both PSII and PSI (Fig. 7; cluster F) reinforces findings described in tobacco plants, suggesting PsbN is required for the biogenesis of both PSI and PSII^47^.

The classical photosynthetic reaction is complete, when chemical energy in the form of ATP and NAD(P)H produced during light-dependent reactions is used to incorporate inorganic carbon (CO_2_) into organic backbones. This reaction is catalyzed by the RuBisCo complex. RuBisCo is composed of several copies of two subunits encoded by *slr0012* (RbcL-large subunit), and *slr0009* (RbcS-small subunit); its assembly is mediated by the product of *slr0011* (RbcX), annotated as a “possible RuBisCo chaperon”^48^. Our results show that genes of the RuBisCo operon belong to a large cluster formed by multiple genes coding for proteins involved in CO_2_ assimilation, including subunits of the NDH-1 complex (Fig. S9)^49^. Nevertheless, notable differences in expression patterns between well-known CO_2_ regulated genes and the RuBisCo operon exist under some conditions, such as iron or sulfate limitation, suggesting that distinct regulatory factors are influencing the expression of these genes under specific conditions (Fig. S9). This suggests that regulation of RuBisCo is primarily modulated by cellular CO_2_ concentration, rather than by the status of other photosynthetic complexes. Therefore, we propose that the regulatory factors behind regulation of the light-independent photosynthetic reaction are distinct from those of the main players involved in light-dependent photosynthetic reactions.

Another application of the integrated *Synechocystis* transcriptome is the elucidation how the different components of the photosynthesis apparatus are coordinated with each other and with other cellular processes (Table S5). In fact, we found various examples of strongly correlated gene pairs that indicate functional connections between photosynthesis and other functional categories. For instance, *sll1184* encoding the heme oxygenase (ho1), which is associated with the functional category ‘Cobalamin, heme, phycobilin and porphyrin, is strongly correlated (median correlation = 0.72) with the functional sub-category PBS. Such a strong correlation is not unexpected, since Ho1 is a key enzyme in the synthesis of the chromophore part of the phycobilisomes^50^. Besides heme-oxygenase other 6 genes part of the category “Cobalamin, heme, phycobilin and porphyrin” show a significant correlation with this category, indicating the existence of common regulatory factors that will aim to ensure an adequate supply of chromophore to the protein scaffold. Similar connections were established between genes encoding PSI subunits, i.e., *slr0737* (*psaD*), PSII and the PBS category. Besides the known functional connection between PSI, PSII and PBS^51^,^52^, this indicates tightly coordinated regulation in the assembly of light-dependent complexes.

Although examples described here demonstrate the value of an integrated *Synechocystis*’ transcriptome, it is important to acknowledge that, caution is required in the interpretation of results from meta-analysis, such as the one presented here. Firstly, the expression datasets were generated in different laboratories using different *Synechocystis* wild-type strains. Additionally, so called “normal” or “standard” conditions have variations between different experiments (Table S6). To minimize the influence of such variations, as well as other potential latent variables, we formulated statistical linear models for the reference designs applied in the original studies and derived well-defined expression contrasts. However, non-linear effects, such as those observed in epistasis, would not be fully captured by this approach and may contribute to the observed variation in gene expression. Despite this, the variation between experimental set-ups can help us to assess the fidelity of observed co-expression patterns. For instance, patterns that are observed across many different experiments and platforms can be considered as more robust, and tend to be more biologically meaningful^6^,^53^. Nevertheless, critical inspection is warranted, especially when conclusions are mainly based on a small subset of the collected transcriptome data.

Therefore, to facilitate visualization and critical inspection of the integrated *Synechocystis*’ transcriptome, we implemented CyanoEXpress (http://cyanoexpress.sysbiolab.eu.), a database with a web-interface based on the GeneXplorer software^54^. It enables other researchers to carry out similar investigations, as we presented here for PSII. CyanoEXpress includes all collated and integrated genome-wide expression data, as well as information regarding samples, their origins, and associated publications. It can assist researchers in the characterization and functional annotation of genes using the guilty-by-association principle, and might also be helpful to determine the underlying regulatory networks. We anticipate that our meta-analyses together with the CyanoEXpress database will contribute to a better understanding of regulation of photosynthesis and its coordination with other processes on a systems level, as well as help to establish *Synechocysti*s as a photosynthetic model organism for systems and synthetic biology studies.

## Methods

### Microarray data collection

Genome-wide expression data for *Synechocystis* were retrieved from three data repositories: NCBI Gene Expression Omnibus (GEO) (22 datasets), KEGG Expression database (13 datasets), and EBI ArrayExpress (2 datasets). These datasets were generated using seven distinct platforms, designated either by their commercial name (CyanoCHIP)^4^ or by the first author whom described their design in the literature (i.e., Postier, Tu, Singh, Zhang, Georg, and Dickson)^16^,^55^,^56^,^57^,^58^,^59^. These datasets comprise measurements of expression changes caused by variations in different environmental factors, including light intensity and quality (UV-B, red versus blue light), temperature, availability of nutrients (deprivation of CO_2_, SO_4_^2−^, Cu^2+^, Fe^2+^; excess of Fe^2+^, Zn^2+^, and presence of Cd^2+^), concentration of atmospheric O_2_, induced oxidative stress (through H_2_O_2_, DCMU, DBMIB, or methyl viologen), and osmotic stress (through NaCl, or sorbitol). Our analysis is not limited to experiments only using WT *Synechocystis,* but also includes microarray measurements that compared the gene expression of mutants with WT after different treatments. Such datasets were mainly derived from two-color microarrays (N = 29), with the exception of six datasets using Agilent^58^ or NimbleGen microarrays^59^. The different microarray platforms are described in more detail in the Supplementary Text. Protein-coding gene names, description and functional categories were extracted from Cyanobase (http://genome.kazusa.or.jp/cyanobase/)^60^.

### Data normalization and differential expression analysis

Microarray data were pre-processed and analyzed using R statistical language and various Bioconductor packages. All dual-channel microarray datasets were individually imported and normalized by optimized intensity-dependent normalization (OIN), based on iterative regression of log fold changes with respect to average log spot intensities^61^. Single-channel microarrays were analyzed using functions from the *limma* package, i.e., the normexp function for background correction, and quantile normalization to adjust signal intensities for each experiment. Intensities from spots corresponding to the same genomic feature were averaged to obtain a value per gene per array. For each of the collected microarray experiments, a linear model was designed based on the sample annotation and evaluated using the lmFit function^62^. The use of explicit models allowed us to define stringent statistical contrasts, which were evaluated for each experiment. These contrasts facilitated comparison of samples for a defined condition (perturbation) with those for the reference condition. For instance, a contrast arises through comparison of gene expression level of a culture grown under high-intensity light compared with one grown under “normal” conditions measured by replicate arrays. Generally, measurements of WT *Synechocystis* under “normal” conditions were used as a reference. It should be noted that these so called “normal” or “standard” can vary between experiments. Table S6 provides an overview of the normal and treatment conditions for the different studies included in the database. Time spans for time-series experiments ranged from 15 min to 4 d, after perturbation. Measurements at individual time points were retained to maximize information, rather than averaging over each time series, and samples at time point 0 were used as reference. Altogether, 115 contrasts were defined for comparisons involving only WT *Synechocystis* gene expression, while 73 contrasts were defined for comparisons between mutant and WT *Synechocystis* gene expressions.

### Hierarchical clustering

For hierarchical two-way clustering (i.e., simultaneous clustering of genes and samples), we used the software Cluster 3.0^63^. Clustering was based on complete linkage, using Spearman Rank correlation as a similarity measure. We carried out clustering only for genes, for which expression values were present for at least 80% of the contrasts. Clusters were visualized using Java TreeView software^64^. Differential expression was displayed as heat maps, using the color range from green to red, with shades of green indicating negative, and shades of red indicating positive expression changes with respect to the reference sample. No differences in expression are indicated by black, while grey represents missing data. Dendrograms attached to the heat maps display the cluster structure, with the length of the branches used as a measure of the similarity between genes, based on their expression. For the statistical enrichment analysis, we used the hypergeometric test (an equivalent of Fisher´s exact test), as implemented in R.

### Effects of environmental conditions on expression of molecular functions

To evaluate whether genes associated with a certain functional category (as defined in Cyanobase, and reproduced in Table S2) show characteristic expression changes for similar environmental conditions, we grouped microarray experiments (and the corresponding expression contrasts) into 12 classes based on their perturbation type (e.g., Fe limitation, oxidative stress, CO_2_ limitation). Experiments performed with mutant strains were excluded from this analysis. Datasets per class are listed in Table S1. For statistical evaluation, the mean gene expression value was calculated for genes linked to the same molecular function (such as PBS). A minimum number of five genes per functional category were required to include a category in this analysis. The individual contrasts (derived earlier in the differential gene expression analysis) were ranked according to their mean expression values. Subsequently, a Wilcoxon rank sum test was applied to assess whether the contrasts associated with each class showed a trend towards high or low expression level within the ranked microarrays. Resulting p-values were converted to false discovery rates (FDR) through the Benjamini-Hochberg procedure. Subsequently, FDR were used to cluster both functional categories and perturbation classes. To perform clustering, “significance vectors” were constructed for each category, with values ranging from −1 to + 1 for each perturbation class. The values were calculated as follows: FDR corresponding to repression were multiplied by (−1) and added to (−1), whereas FDR corresponding to activation were subtracted from (+1). Thus, perturbations that led to a highly significant activation are represented by values close to (+1), while perturbations that led to a highly significant repression are represented by values close to (−1). These significance vectors were merged into a matrix and a two-way hierarchical clustering of functional categories and perturbation classes was carried out.

### Variability of gene expression within sub-categories

To assess the variability of gene expression within different sub-categories, we calculated the standard deviation of expression of each gene. Subsequently, the average was computed for all genes associated with a particular sub-category in Cyanobase. The statistical significance of the observed values was obtained through comparison with the expected normal background distribution based on 1000 randomized gene sets of the same size. P-values were calculated from the z-scores of the observed average values and converted to FDR.

### Co-expression network of functional categories

A co-expression network of functional categories was generated by first calculating the Spearman correlation for each pair of genes based on the integrated expression data (Table S7). This gene network was then converted into a co-expression network of functional sub-categories defined in Cyanobase. For this purpose, the mean correlation between genes included in the same category was computed for each category. Similarly, the mean correlation was computed for all possible pairs of functional categories by averaging over the correlation of corresponding gene pairs, i.e., the correlation of genes associated with different categories. To obtain a measure for the strength of co-expression, the z-scores for the mean correlation values within and between categories were calculated based on the mean and standard deviation of the total set of observed correlations. Additionally, log odds were calculated for the presence of highly correlated gene pairs among all gene pairs within a category or between categories. Gene pairs were considered highly correlated, if their correlation coefficient was among the top 10% of all correlation coefficients. To generate Fig. 6, only edges with a z-score of greater than 7 were retained, resulting in a network of 25 nodes and 47 edges.

### Detection of co-expression of genes associated with different functional categories

To detect genes, that were highly transcriptionally co-regulated with gene associated with 8 functional sub-categories of ‘Photosynthesis and Respiration’ (i.e. ‘Atp synthase’,‘CO_2_ fixation’, ‘Cytochrome *b*_6_ *f* complex’, ‘NADH dehydrogenase’, ‘PSI’, ‘PSII’, ‘PBS’ and ‘Respiratory terminal oxidases’), we compared the median pair-wise correlation between genes. More specifically, we calculated first the median correlation of genes within one of the eight specific sub-categories with other genes of the same sub-category and then derived a threshold, so that 80% of the calculated correlation coefficients were below. This leads to individual thresholds for the sub-category indicating their internal co-expression. For ‘CO_2_ fixation’ and ‘NADH dehydrogenase’, a higher threshold (90%) was set, since the internal co-expression tend to be relatively weak. Subsequently, median pair-wise correlation was calculated for each *Synchocystis* gene with genes of the eight selected sub-categories and compared to the relevant thresholds. Genes whose correlation passed the thresholds can be found in Supplementary Table S5.

## Additional Information

**How to cite this article**: Hernández-Prieto, M. A. *et al.* The Transcriptional Landscape of the Photosynthetic Model Cyanobacterium *Synechocystis* sp. PCC6803. *Sci. Rep.* **6**, 22168; doi: 10.1038/srep22168 (2016).

## Supplementary Material

Supplementary Information

Supplementary Table S1

Supplementary Table S3

Supplementary Table S5

Supplementary Table S6

## Figures and Tables

**Table 1:**
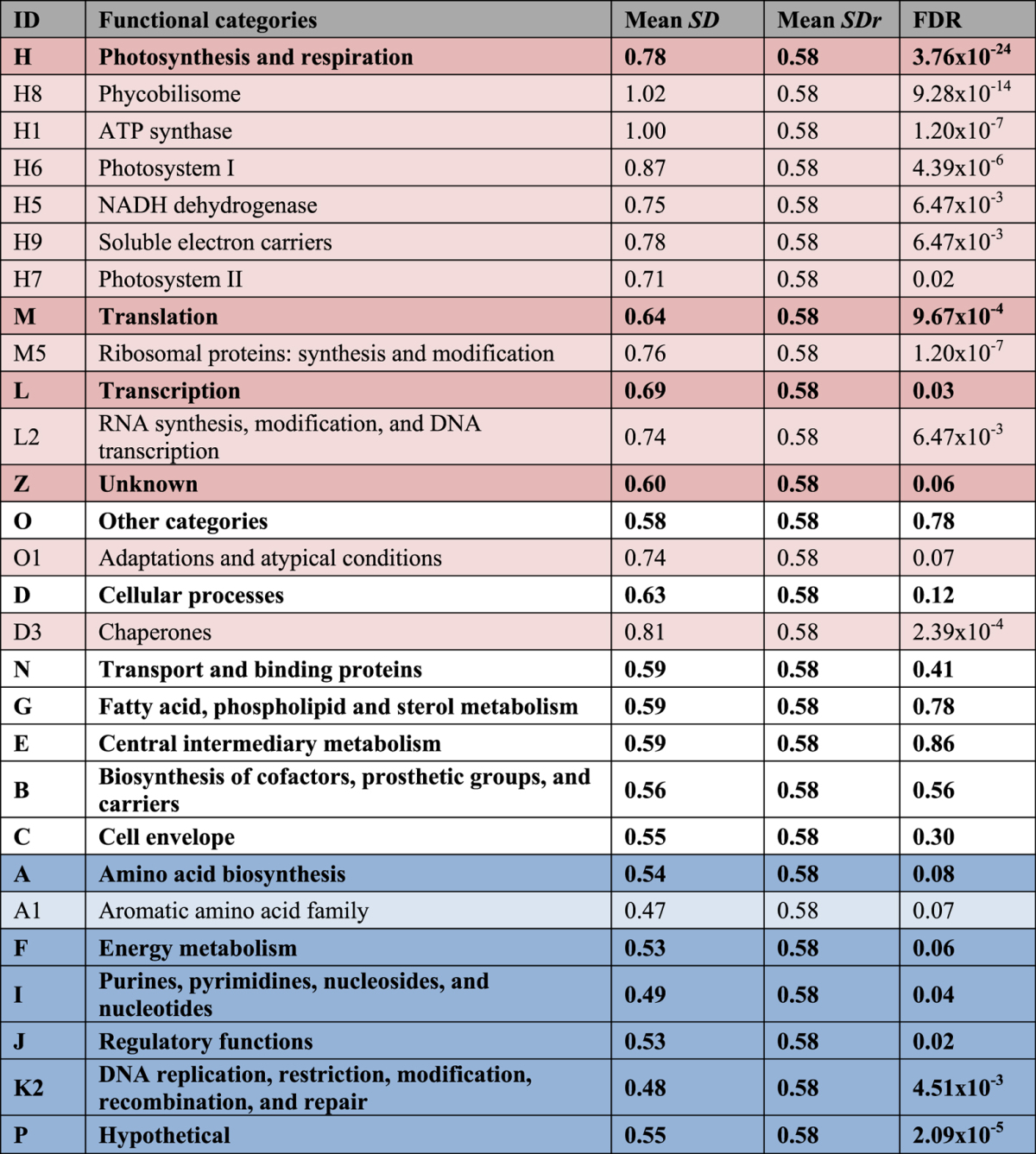
Variability of gene expression in different functional categories.

This table lists the average standard deviation (*Mean SD*) of expression for genes associated with a particular functional category and its statistical significance (FDR) based on the comparison with the average standard deviation (*Mean SDr*) for random gene samples of the same size. Categories whose genes show significantly more variable expression than expected by chance are marked by shades of red (FDR < 0.01).; categories whose genes are significantly less variable than expected by chance are marked in shades of blue. Bold font indicates main functional categories, which were ordered, so that categories with significantly larger variability were placed higher in the table, while categories with significantly smaller variability were located at the bottom. While all main categories are displayed, sub-categories were only shown if they were statistically significant. The results for the entire list of 70 sub-categories can be found in Supplementary Table S5, available online.
