# Supplementary material for: The Transcriptional Landscape of the Photosynthetic Model Cyanobacterium Synechocystis sp. PCC6803
Source: Sci Rep. 2016 Feb 29;6:22168. doi: 10.1038/srep22168 (PMC4770689; doi:10.1038/srep22168)
Supplement: Supplementary Information [file srep22168-s1.pdf]

**Supplementary Information**

# The Transcriptional Landscape of the Photosynthetic Model Cyanobacterium *Synechocystis* sp. PCC6803

Miguel A. Hernández Prieto, Trudi Ann Semeniuk, Joaquín Giner-Lamia, Matthias E. Futschik

## Contents:

1. Description of microarray platforms 2

2. Quality control of individual datasets 3

Figure S1 4

Figure S2 5

Figure S3 5

Figure S4 6

3. Detection of outlier experiments 7

Figure S5 7

Figure S6 8

4. Other supplemental figures

Figure S7 9

Figure S8 10

Figure S9 11

Figure S10 12

5. Supplemental tables

Table S1 13

Table S2 14

Table S3 17

Table S4 18

Table S5 21

Table S6 22

Table S7 23

6. Supplemental references 26

## *1. Description of microarray platforms*

Publicly available microarray expression data for *Synechocystis* that have been integrated in our study, were generated using seven different platforms, of which three platforms (CyanoCHIP, Postier, and Tu) contain complementary sequences to open reading frames (ORFs) produced by polymerase chain reaction (PCR), while the rest of the platforms use synthetically generated oligomers as probes. Here, we provide short descriptions of these platforms based on their original descriptions by the authors.

**CyanoCHIP**: The CyanoCHIP platform is the most commonly used platform because of its early development and commercial availability[1]. The probes spotted on this type of microarray contained products generated by PCR, using up to 1-kb of the C-terminal coding regions of long ORFs and the full-length of shorter ORFs. In total, it contains duplicated probes for 3079 ORFs, covering 93% of the ORFs located in the chromosome of *Synechocystis*. In addition to these probes, another 48 spots are included as internal controls (3 spots), a negative control (1 spot), and as positional markers (44 spots).

**Postier:** The platform by Postier et al.[2] was generated by adding an extra PCR step, using primers complementary to a universal sequence introduced into the primers used for the first PCR. In total, this microarray slide contains 3163 triplicated probes of different lengths, covering 95% of the individual *Synechocystis* chromosomal ORFs.

**Tu**: This platform was generated using the same protocol as in Postier et al. However, it included 70-mer oligonucleotides specific for 67 genes, for which amplification failed by PCR. It contains probes for 3165 genes (2641 in quadruplicates, and the remainder with even higher numbers of replicates) [3].

**Zhang:** The microarray designed by Zhang et al.[4] contains 70-nt-long oligomers probes per gene, covering 3064 ORFs from the *Synechocystis* chromosome, spotted onto poly-L-lysine coated microscopic glass slides.

**Singh:** The *Synechocystis* 11K oligo DNA microarray designed by Singh et al.[5] comprises 60-nt-long oligomers, spotted on an Agilent support. It covers 3459 genes, including genes coded in the mega-plasmids.

**Georg:** The platform designed by Georg et al.[6] contains 42303 probes, with lengths between 45- to 60–nt, spotted on an 44K Agilent custom microarray format having 15951 duplicates, 321 triplicates, and the remaining probes with a greater number of replicates. Of these probes, 21022 were directed against all 3317 chromosome-located *Synechocystis* genes, as well as 82 of the 408 plasmid-located genes annotated in Cyanobase[7]. The microarray platform also comprises 5028 probes for 1875 intragenic elements, 1939 probes against antisense-RNAs, and probes for 608 small-RNAs originating from intergenic regions.

**Von Wobeser:** The design of this platform was assisted by the software Array Designer 2.0 (Premier Biosoft International). The platform contains 15208 probes against *Synechocystis* genes consisting of one to four probes per gene. The probes were custom printed on an Agilent 8x15K custom designed support using SurePrint technology[8].

**<u>Dickson:</u>** The most recent microarray platform developed for *Synechocystis* is the 60-oligomer array described by Dickson et al.[9] and synthesised by NimbleGen containing 13 probes per target (385,000 probes) directed against 3567 protein-coding genes.

## *2. Quality control of individual datasets*

To calculate differential expression for the various datasets in a standardized manner, all datasets were loaded individually as marrayRaw objects in R/Bioconductor. Data upload and conversion were carried out using R scripts customized for each platform and repository format. Subsequently, datasets were inspected for artefacts using various tools.

Firstly, we assessed the intensity distribution of microarrays within each dataset. An example of this process is shown in Figure S2, which was produced using the data generated by Rowland *et al.*[10] studying thermal acclimation (the raw microarray data can be accessed through GEO accession: GSE21133.) In this study, the researchers used the two-color CyanoCHIP platform containing probes against 3079 Synechocystis 6803 genes. Their time series included four replicates for each of the five time points, with the control sample (t = 0) labelled with Cy3 and the test samples (t = 30 / 60/ 120 / 240 / 480 mins) with Cy5.

To compare the distribution of the $log_2$ – transformed signal ratios (M) for each microarray, box-plots were generated for raw and normalized data. Box-plots facilitate the visualization of data heterogeneity and serve to corroborate that the applied normalization method (i.e., OIN) corrected the observed intensity biases. As seen in Figure S2A, the distribution of probe intensities differs noticeably between replicates before normalization. Such differences are indicative of inter-sample variation, which can be caused by various experimental factors. In this case, normalization using the OIN algorithm corrected these observed scaling discrepancies (Figure S2B).

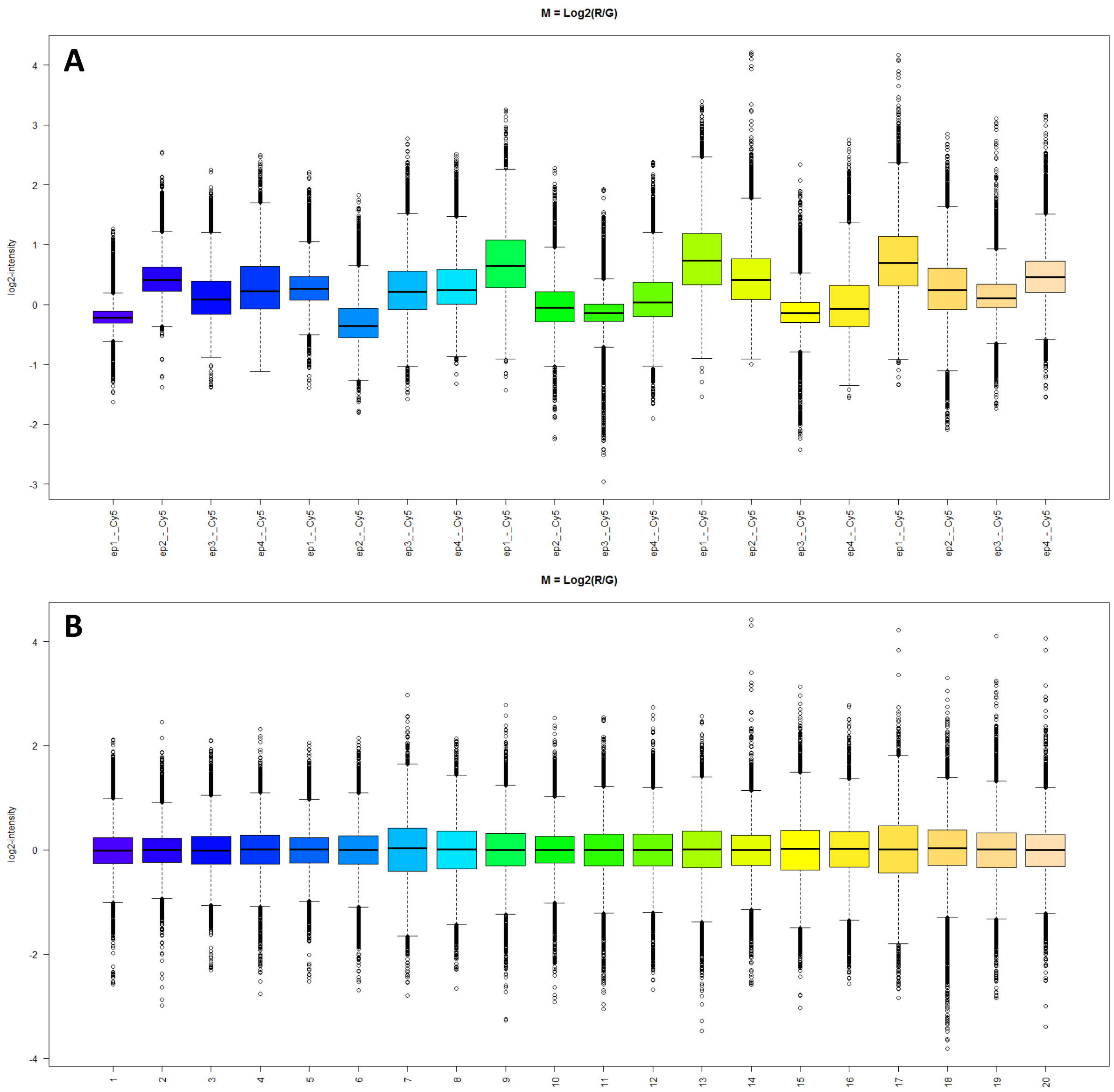


**Figure S1**. Boxplots comparing the distribution of the pre-normalized (top; A) and post-normalized (bottom; B) $\log_2$ signal ratios (M) between microarrays in the Rowland et al. (2010) dataset. R/G = red/green

Alternatively, the spread of the signal intensities was visualized using density plots. For two-color arrays, intensities for each channel were plotted independently. In this case, the x-axis represents the intensities of the probes, and the y-axis shows density level (Figure S3). These plots can be indicative of artefacts and interferences linked to high background signals. As observed previously with the box-plots, the normalization efficiently reduced the observed differences in such distributions (Figure S3B).

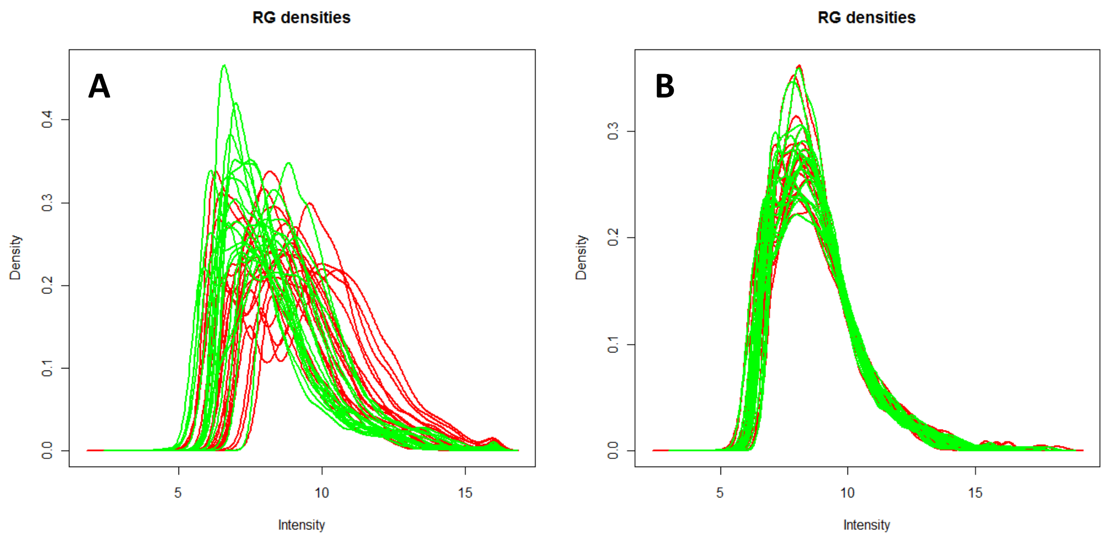


**Figure S2.** Raw (A) and normalized (B) probe intensity values plotted against their density on the arrays. Green curves represent the control probes (Cy3-labeled), while red curves represent the test samples labelled with Cy5. RG = red green.

For datasets generated by two-color arrays, we also used M-A plots to investigate dye bias and to determine whether normalization corrected it (Figure S4).

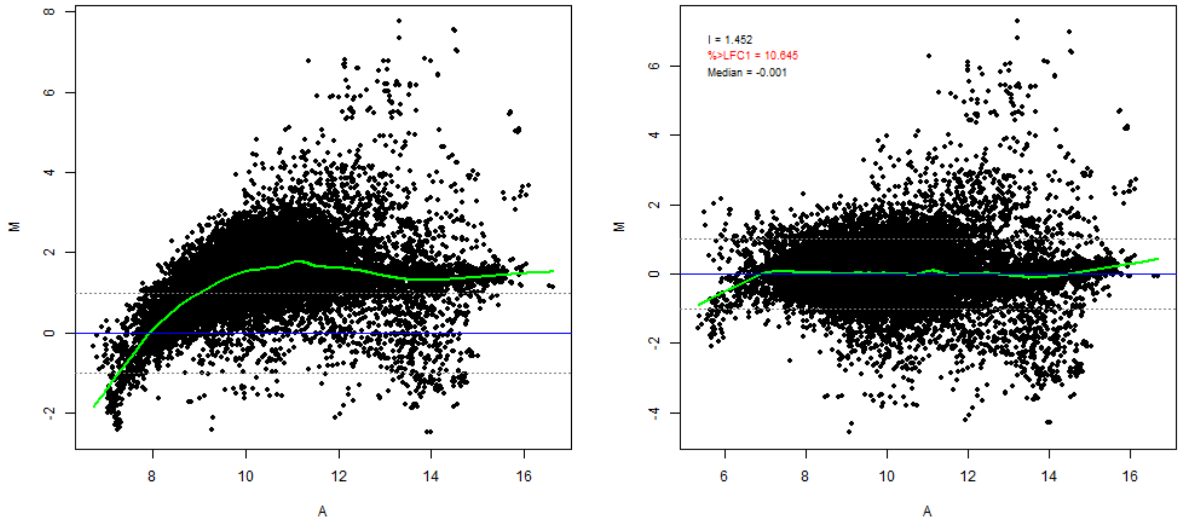


**Figure S3:** Plots of logged fold changes (LFC) with respect to average logged spot intensity of both channels (Cy3, Cy5) for raw data (left panel) and normalized data (right panel). Raw data show a clear bias towards Cy5 intensities (indicated by M values larger than zero) at higher intensities, whereas this dye-bias is corrected in the normalized data.

Finally, scatter-plots of the logged intensities for each channel extracted from the different replicates served to visualize the experimental reproducibility (Figure S5; top panel). To examine the result of

normalization on replicate bases, a plot was generated using the normalized M values (Figure S5; lower panel).

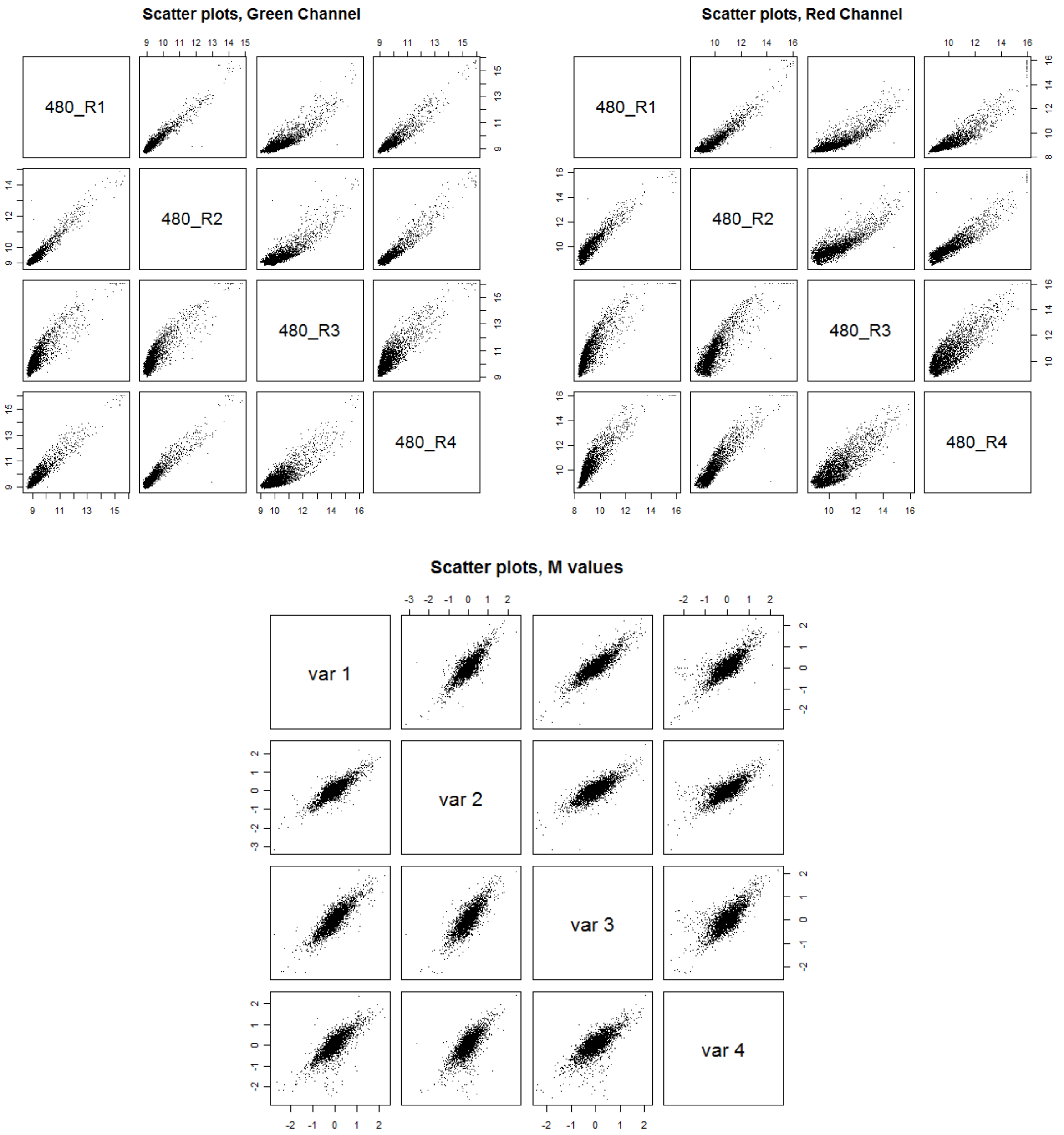


**Figure S4:** Scatter plots of logged intensities for both Cy3(green)- and Cy5(red)-channels (top panel) and normalized logged fold changes (bottom panel).

## 3. Detection of outlier experiments

In microarray analyses, we generally assume that only a small fraction of genes is differentially expressed. Thus, we expect a similar overall distribution of different expression in the collected datasets, despite being generated by various laboratories using different platforms and protocols. To check whether this is indeed the case, and whether some experiments or samples are distinct from other experiments or samples, we applied hierarchical clustering and principal component analysis (PCA) to the expression changes of the integrated data set. Hierarchical clustering of microarray data indicated that the samples from an unpublished data set (GSE5391) had quite distinct expression, forming a clearly separated cluster (Figure S6). This was also reflected in the PCA results, where these samples showed considerable separation from the remaining samples (Figure S7). Although such analyses do not per se demonstrate that samples from GSE5391 are less reliable, they nevertheless show that these samples are outliers. To avoid effects of a small number of outliers, we decided to remove this dataset from our analyses. In addition, it has not been described in any publication. Two other non-published datasets GSE4604, and GSE4613, for which sample descriptions did not match with the clustering of their replicates also were removed.

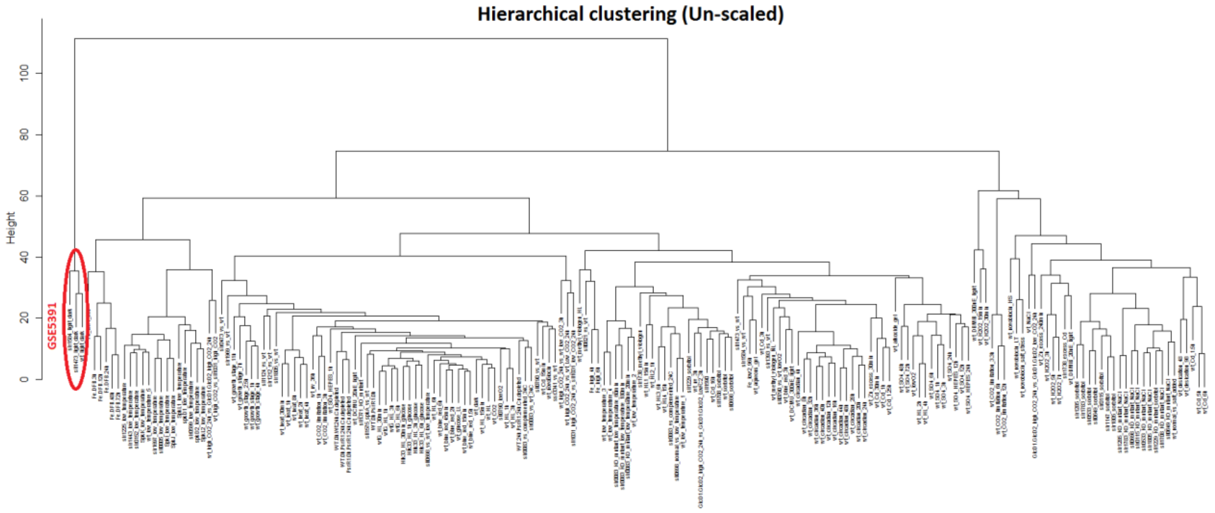


**Figure S5:** Dendrogram showing the hierarchical clustering of 177 contrasts from 33 independent experiments based on their normalized M values. Clustering was carried out on average linkage and complete correlation. Samples from data set GSE5391 are highlighted, showing their outlier status.

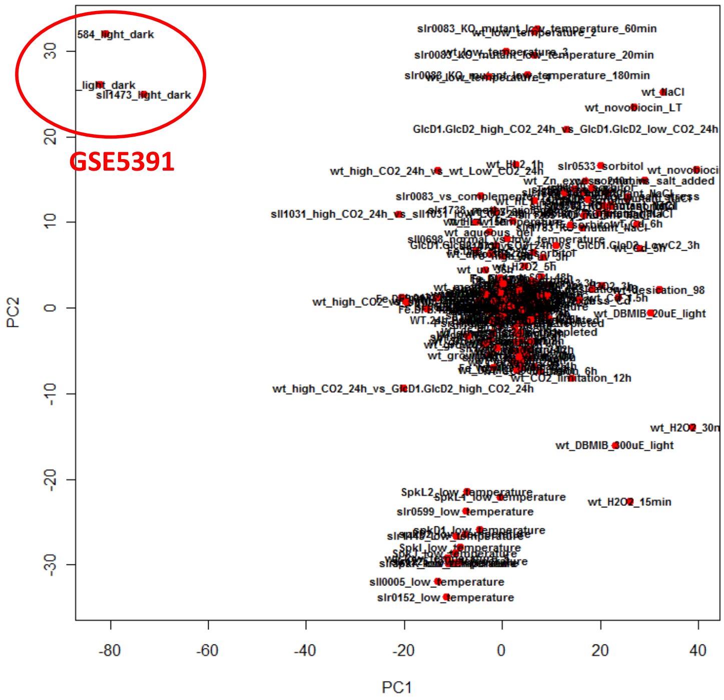


**Figure S6:** Principal component analysis of all datasets. The two first principal components are shown for 177 contrasts, arising from 33 independent studies. Samples from GSE5391 are highlighted, emphasising their outlier status.

## *4. Other supplemental figures*

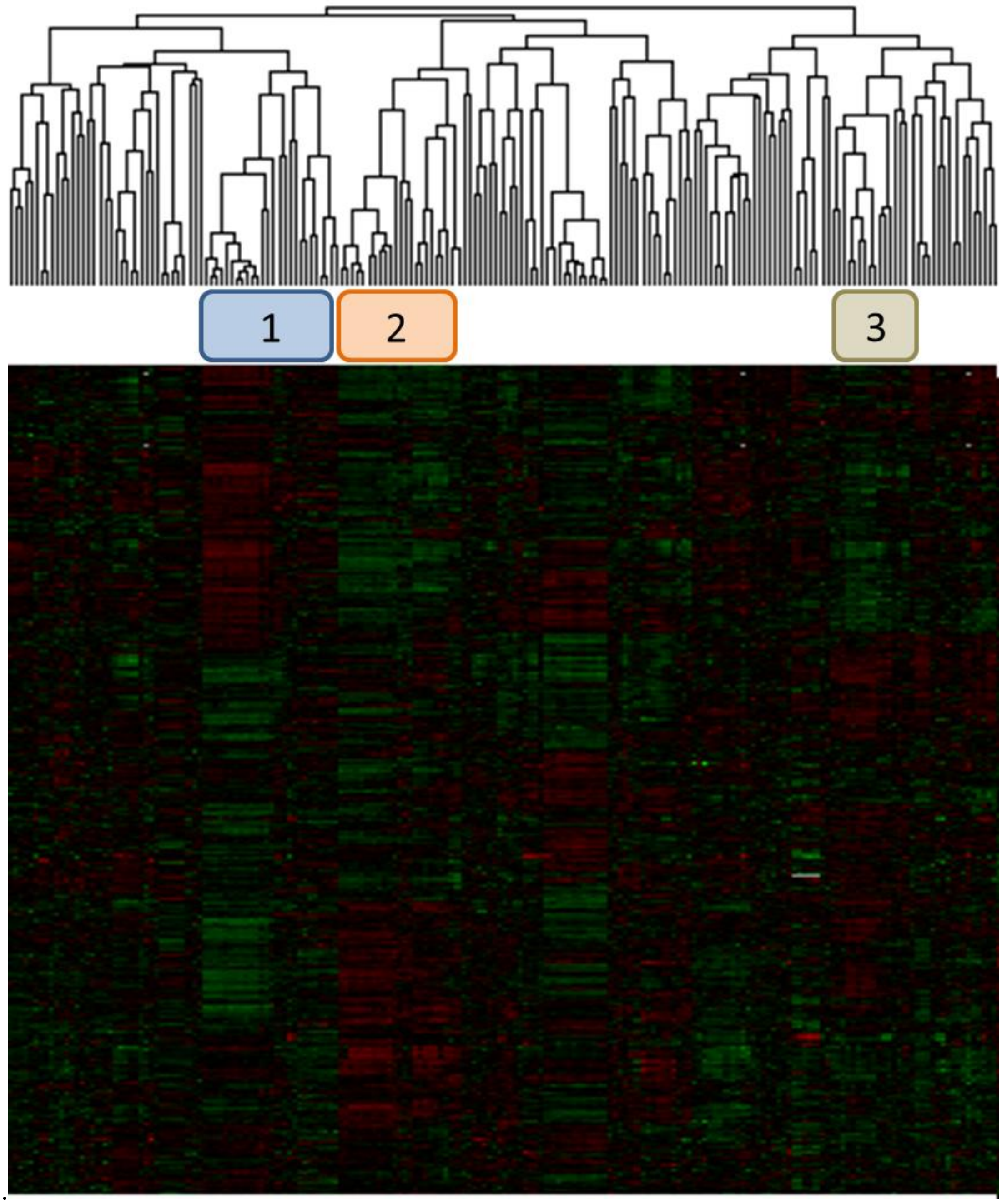


**Figure S7:** Heat map showing clustering of the contrast obtained using wild type or mutated strains analyzed in this study. Experiments performed using mutants lacking genes encoding Ser/Thr protein kinases[11], or genes encoding histidine kinases, or its cognate response regulator[12,13] are labeled by boxes numbered from 1 to 3, respectively.

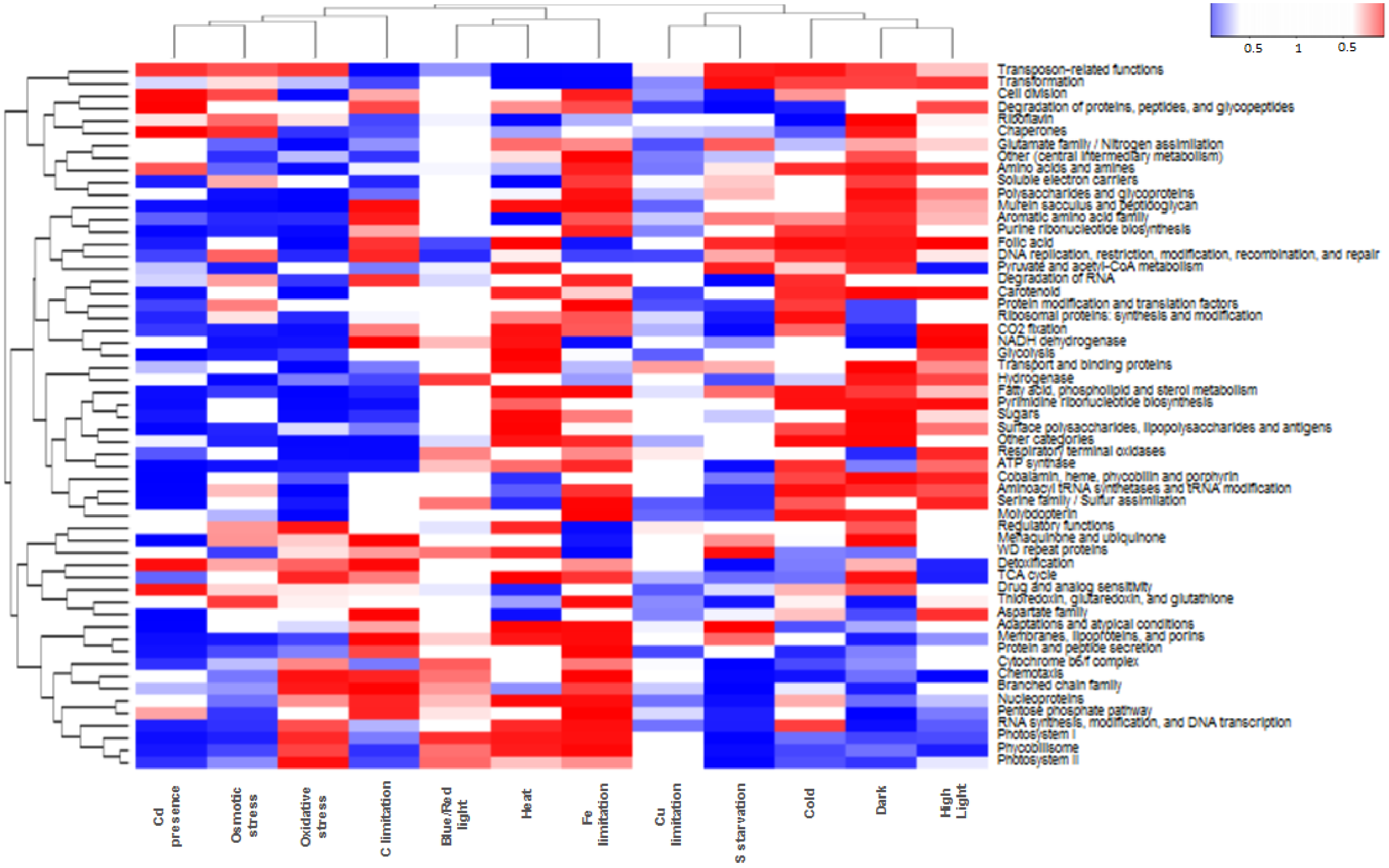


**Figure S8:** Graphical representation of adaption of molecular processes to different environmental stimuli. The heat map shows activation (as shades of red) and repression (as shades of blue) of *Synechocystis* genes grouped within cellular functional sub-categories (y-axis) under different types of environmental perturbations (x-axis). The color intensities (see color bar) are defined by the calculated significance expressed as False Discovery Rates, outlined in Materials and Methods. Darker shades indicate higher significance of transcriptional changes.

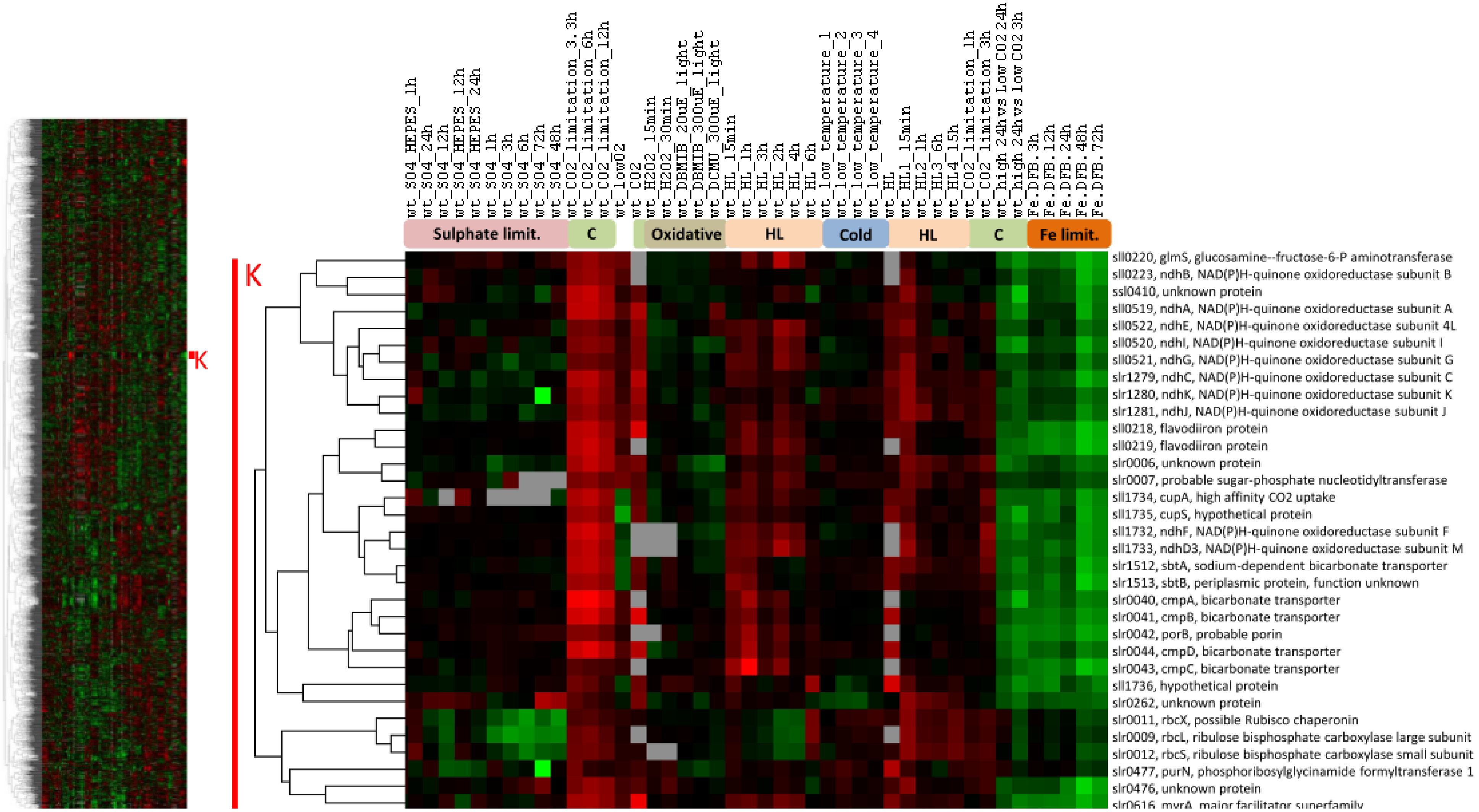


**Figure S9:** Clustering of genes with expression profiles resembling the expression of genes encoding the two subunits of the RuBisCo enzymatic complex. The cluster is enriched in a large number of genes encoding proteins related to $CO_2$, including subunits of the NDH-I complex. The cluster arose from the comparison of all environmental samples. The heat map obtained from such a comparison (discussed in Figure 2) is shown as a thumbnail image on the left, with a red bar indicating the location of the cluster shown on the right. Note, that some contrasts were trimmed to highlight distinct expression patterns; the complete expression patterns for the genes are available on the CyanoEXpress website (http://cyanoexpress.sysbiolab.eu/).

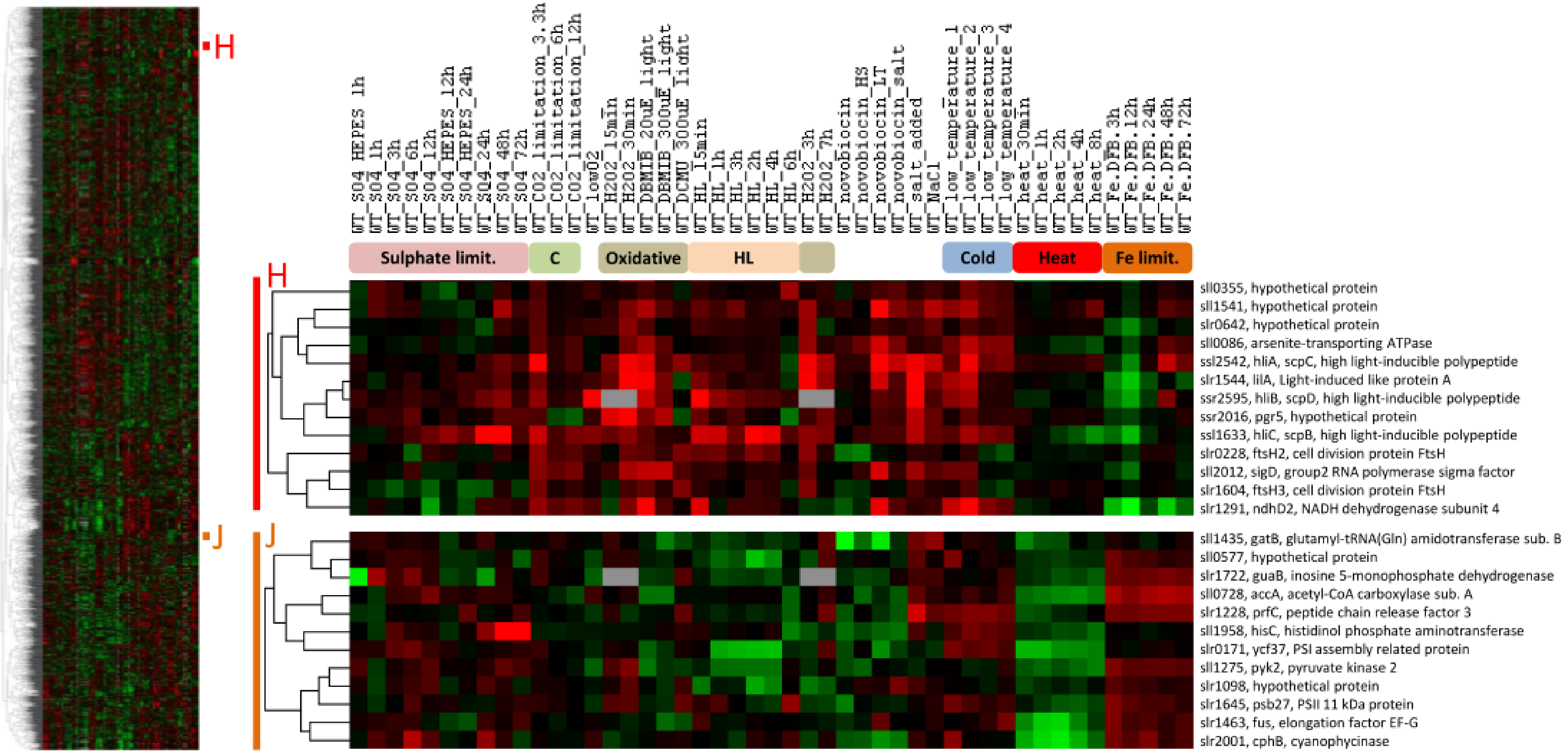


**Figure S10:** The set of clusters containing genes encoding proteins involved in the assembly of the photosystems. Cluster H groups the stress-induced genes encoding high-light intensity proteins (HLIPs), and the FtsH proteases. Cluster J encompasses the genes encoding Ycf37 and Psb27 that have been related in the literature to the assembly of both photosystems. The clusters arose from the comparison of all the samples obtained using wild-type strains grown under different environmental perturbations. The heat map obtained from this comparison (discussed in Figure 2) is shown as a thumbnail image on the left. The approximate location and size of the clusters discussed are indicated by colored bars. Some conditions were trimmed for publication; the full image is available on the CyanoEXpress website (http://cyanoexpress.sysbiolab.eu/).

## *5. Supplemental tables*

**Table S1:** Summary of relevant information on microarray data used in this study. Excel file listing the transcriptome datasets and measurements integrated and examined in the meta-analysis.

**[Supplementary Table S1 online]**

**Table S2:** Functional categories and subcategories represented in Cyanobase. The functional enrichment analysis of gene clusters, as well as the prediction of gene functions is based on this classification system. The list of genes in each category can be obtained from Cyanobase (http://genome.kazusa.or.jp/cyanobase/).

| Category ID | Functional Category | Subcategory ID | Functional Subcategory |
|---|---|---|---|
| A | Amino acid biosynthesis | A1 | Aromatic amino acid family |
| | | A2 | Aspartate family |
| | | A3 | Branched chain family |
| | | A4 | Glutamate family / Nitrogen assimilation |
| | | A6 | Serine family / Sulfur assimilation |
| B | Biosynthesis of cofactors, prosthetic groups, and carriers | B1 | Biotin |
| | | B2 | Folic acid |
| | | B3 | Cobalamin, heme, phycobilin and porphyrin |
| | | B4 | Lipoate |
| | | B5 | Menaquinone and ubiquinone |
| | | B6 | Molybdopterin |
| | | B7 | Pantothenate |
| | | B8 | Pyridoxine |
| | | B9 | Riboflavin |
| | | B10 | Thioredoxin, glutaredoxin, and glutathione |
| | | B12 | Thiamin |
| | | B13 | Carotenoid |
| | | B15 | Quinolinate |
| | | B17 | Others |
| | | B18 | Nicotinate and nicotinamide |
| C | Cell envelope | C1 | Membranes, lipoproteins, and porins |
| | | C2 | Murein sacculus and peptidoglycan |
| | | C3 | Surface polysaccharides, lipopolysaccharides and antigens |
| | | C4 | Surface structures |
| D | Cellular processes | D1 | Cell division |
| | | D2 | Cell killing |
| | | D3 | Chaperones |
| | | D4 | Detoxification |
| | | D5 | Protein and peptide secretion |
| | | D6 | Transformation |
| | | D7 | Chemotaxis |
| E | Central intermediary metabolism | E1 | Amino sugars |
| | | E3 | Other |

| | | | |
|---|---|---|---|
| | | E4 | Phosphorus compounds |
| | | E6 | Polysaccharides and glycoproteins |
| F | Energy metabolism | F2 | Amino acids and amines |
| | | F9 | Glycolysis |
| | | F10 | Pentose phosphate pathway |
| | | F11 | Pyruvate dehydrogenase |
| | | F12 | Sugars |
| | | F13 | TCA cycle |
| | | F14 | Pyruvate and acetyl-CoA metabolism |
| | | F15 | Glycolate pathway |
| G | Fatty acid, phospholipid and sterol metabolism | G | Fatty acid, phospholipid and sterol metabolism |
| H | Photosynthesis and respiration | H1 | ATP synthase |
| | | H2 | CO2 fixation |
| | | H3 | Cytochrome b6/f complex |
| | | H5 | NADH dehydrogenase |
| | | H6 | Photosystem I |
| | | H7 | Photosystem II |
| | | H8 | Phycobilisome |
| | | H9 | Soluble electron carriers |
| | | H10 | Respiratory terminal oxidases |
| I | Purines, pyrimidines, nucleosides, and nucleotides | I2 | Interconversions and salvage of nucleosides and nucleotides |
| | | I3 | Purine ribonucleotide biosynthesis |
| | | I4 | Pyrimidine ribonucleotide biosynthesis |
| J | Regulatory functions | J | Regulatory functions |
| K2 | DNA replication, restriction, modification, recombination, and repair | K2 | DNA replication, restriction, modification, recombination, and repair |
| L | Transcription | L1 | Degradation of RNA |
| | | L2 | RNA synthesis, modification, and DNA transcription |
| M | Translation | M1 | Aminoacyl tRNA synthetases and tRNA modification |
| | | M2 | Degradation of proteins, peptides, and glycopeptides |
| | | M3 | Nucleoproteins |
| | | M4 | Protein modification and translation factors |
| | | M5 | Ribosomal proteins: synthesis and modification |
| N | Transport and binding proteins | N | Transport and binding proteins |
| O | Other categories | O1 | Adaptations and atypical conditions |

| | | | |
|---|---|---|---|
| | | O3 | Drug and analog sensitivity |
| | | O7 | Other |
| | | O7a | WD repeat proteins |
| | | O7b | Hydrogenase |
| | | O8 | Transposon-related functions |
| P | Hypothetical | P | Hypothetical |
| Z | Unknown | Z | Unknown |

**Table S3:** Excel file including the results of the functional enrichment analysis for the clusters identified in Figure 2. The colors of the column headings correspond with the different clusters shown in Figure 2.

**[Supplementary Table S3 online]**

**Table S4**: Variability of gene expression in functional subcategories. This table lists the average standard deviation (***Mean SD***) of expression for genes associated with a particular functional sub-category, the ***z***-score and the corresponding statistical significance (unadjusted ***p***-value and ***FDR***) based on the comparison with the average standard deviation (***Mean SDr***) for random gene samples of the same size.

| ID | Functional subcategories | Mean *SD* | Mean *SDr* | z | p | FDR |
|---|---|---|---|---|---|---|
| A1 | Aromatic amino acid family | 0.469184 | 0.580801 | -2.48 | 0.013138 | 0.074787 |
| A2 | Aspartate family | 0.566043 | 0.580136 | -0.21107 | 0.832829 | 0.948144 |
| A3 | Branched chain family | 0.552798 | 0.582693 | -0.46567 | 0.641454 | 0.908513 |
| A4 | Glutamate family / Nitrogen assimilation | 0.574076 | 0.580405 | -0.16573 | 0.868369 | 0.959094 |
| A6 | Serine family / Sulfur assimilation | 0.542308 | 0.585563 | -0.5419 | 0.587889 | 0.853015 |
| B1 | Biotin | 0.540336 | 0.577758 | -0.28541 | 0.775329 | 0.935822 |
| B10 | Thioredoxin, glutaredoxin, and glutathione | 0.577389 | 0.58168 | -0.07802 | 0.937809 | 0.977435 |
| B12 | Thiamin | 0.385688 | 0.581352 | -1.66443 | 0.096027 | 0.355301 |
| B13 | Carotenoid | 0.582487 | 0.580585 | 0.025741 | 0.979464 | 0.992881 |
| B15 | Quinolinate | 0.621406 | 0.581845 | 0.253818 | 0.799637 | 0.935822 |
| B17 | Others | 0.403379 | 0.587237 | -1.10139 | 0.270727 | 0.667793 |
| B18 | Nicotinate and nicotinamide | 0.486542 | 0.577297 | -0.82634 | 0.40861 | 0.703189 |
| B2 | Folic acid | 0.503174 | 0.584428 | -0.77027 | 0.441142 | 0.707012 |
| B3 | Cobalamin, heme, phycobilin and porphyrin | 0.609909 | 0.581111 | 0.83363 | 0.404489 | 0.703189 |
| B4 | Lipoate | 0.443248 | 0.582026 | -1.00044 | 0.317095 | 0.682519 |
| B5 | Menaquinone and ubiquinone | 0.513576 | 0.578441 | -0.88131 | 0.378152 | 0.682519 |
| B6 | Molybdopterin | 0.668971 | 0.580893 | 0.928833 | 0.352976 | 0.682519 |
| B7 | Pantothenate | 0.509765 | 0.582586 | -0.61394 | 0.539256 | 0.831353 |
| B8 | Pyridoxine | 0.423676 | 0.579274 | -1.19809 | 0.230883 | 0.596773 |
| B9 | Riboflavin | 0.584974 | 0.575014 | 0.098825 | 0.921277 | 0.973922 |
| C1 | Membranes, lipoproteins, and porins | 0.698236 | 0.578108 | 1.79414 | 0.072791 | 0.299251 |
| C2 | Murein sacculus and peptidoglycan | 0.509012 | 0.582141 | -1.52956 | 0.126126 | 0.424242 |
| C3 | Surface polysaccharides, lipopolysaccharides and antigens | 0.523381 | 0.580754 | -1.35939 | 0.174022 | 0.559896 |
| C4 | Surface structures | 0.448171 | 0.583066 | -1.02352 | 0.306064 | 0.682519 |
| D1 | Cell division | 0.527286 | 0.581508 | -0.89211 | 0.372333 | 0.682519 |
| D2 | Cell killing | 0.628554 | 0.585404 | 0.364163 | 0.715736 | 0.929202 |
| D3 | Chaperones | 0.81904 | 0.581555 | 4.272249 | 1.94E-05 | 0.000239 |
| D4 | Detoxification | 0.623816 | 0.58215 | 0.42016 | 0.674369 | 0.908513 |

| | | | | | | |
|---|---|---|---|---|---|---|
| D5 | Protein and peptide secretion | 0.558664 | 0.584341 | -0.42628 | 0.669906 | 0.908513 |
| D6 | Transformation | 0.483898 | 0.581466 | -1.07158 | 0.283908 | 0.667894 |
| D7 | Chemotaxis | 0.643052 | 0.581831 | 1.060716 | 0.288819 | 0.667894 |
| E1 | Amino sugars | 0.629677 | 0.587946 | 0.175843 | 0.860417 | 0.959094 |
| E3 | Other | 0.439575 | 0.583032 | -1.59018 | 0.111793 | 0.393939 |
| E4 | Phosphorus compounds | 0.598013 | 0.582757 | 0.116966 | 0.906887 | 0.973922 |
| E6 | Polysaccharides and glycoproteins | 0.641153 | 0.579273 | 1.190448 | 0.23387 | 0.596773 |
| F10 | Pentose phosphate pathway | 0.581149 | 0.581388 | -0.00294 | 0.997657 | 0.997657 |
| F11 | Pyruvate dehydrogenase | 0.61337 | 0.581701 | 0.267048 | 0.789432 | 0.935822 |
| F12 | Sugars | 0.517767 | 0.582218 | -1.26137 | 0.207175 | 0.589652 |
| F13 | TCA cycle | 0.480609 | 0.580395 | -1.27776 | 0.201335 | 0.589652 |
| F14 | Pyruvate and acetyl-CoA metabolism | 0.556642 | 0.578297 | -0.26581 | 0.790388 | 0.935822 |
| F15 | Glycolate pathway | 0.488534 | 0.582381 | -0.80958 | 0.418184 | 0.70331 |
| F2 | Amino acids and amines | 0.493672 | 0.580769 | -1.90772 | 0.056428 | 0.260979 |
| F9 | Glycolysis | 0.595455 | 0.580976 | 0.241252 | 0.80936 | 0.935822 |
| H1 | ATP synthase | 1.002484 | 0.576136 | 5.852134 | 4.85E-09 | 1.20E-07 |
| H10 | Respiratory terminal oxidases | 0.504273 | 0.581019 | -0.97858 | 0.327785 | 0.682519 |
| H2 | CO2 fixation | 0.685573 | 0.584551 | 1.695937 | 0.089898 | 0.350128 |
| H3 | Cytochrome b6/f complex | 0.651777 | 0.580748 | 0.944019 | 0.34516 | 0.682519 |
| H5 | NADH dehydrogenase | 0.750307 | 0.581345 | 3.360372 | 0.000778 | 0.006466 |
| H6 | Photosystem I | 0.874666 | 0.579814 | 5.167501 | 2.37E-07 | 4.39E-06 |
| H7 | Photosystem II | 0.707899 | 0.579937 | 2.90835 | 0.003633 | 0.024443 |
| H8 | Phycobilisome | 1.024777 | 0.583569 | 7.99909 | 1.25E-15 | 9.28E-14 |
| H9 | Soluble electron carriers | 0.777154 | 0.577776 | 3.371571 | 0.000747 | 0.006466 |
| I2 | Interconversions and salvage of nucleosides and nucleotides | 0.498022 | 0.580596 | -0.78549 | 0.432164 | 0.707012 |
| I3 | Purine ribonucleotide biosynthesis | 0.494251 | 0.581311 | -1.81117 | 0.070115 | 0.299251 |
| I4 | Pyrimidine ribonucleotide biosynthesis | 0.490322 | 0.582479 | -1.30752 | 0.191035 | 0.589026 |
| K2 | DNA replication, restriction, modification, recombination, and repair | 0.484635 | 0.581373 | -3.22594 | 0.001256 | 0.009291 |
| L1 | Degradation of RNA | 0.51726 | 0.584169 | -0.757 | 0.449048 | 0.707012 |
| L2 | RNA synthesis, modification, and DNA transcription | 0.73915 | 0.580333 | 3.357552 | 0.000786 | 0.006466 |
| M1 | Aminoacyl tRNA synthetases and tRNA modification | 0.562489 | 0.581538 | -0.54608 | 0.585009 | 0.853015 |
| M2 | Degradation of proteins, peptides, and glycopeptides | 0.578716 | 0.5801 | -0.03042 | 0.97573 | 0.992881 |

| | | | | | | |
|---|---|---|---|---|---|---|
| M3 | Nucleoproteins | 0.65469 | 0.578234 | 0.913926 | 0.360756 | 0.682519 |
| M4 | Protein modification and translation factors | 0.576037 | 0.580521 | -0.10495 | 0.916413 | 0.973922 |
| M5 | Ribosomal proteins: synthesis and modification | 0.758861 | 0.58195 | 5.898047 | 3.68E-09 | 1.20E-07 |
| O1 | Adaptations and atypical conditions | 0.73945 | 0.577776 | 2.446498 | 0.014425 | 0.076247 |
| O3 | Drug and analog sensitivity | 0.566843 | 0.580679 | -0.28695 | 0.77415 | 0.935822 |
| O7 | Other | 0.557736 | 0.581795 | -1.22737 | 0.219683 | 0.596773 |
| O7a | WD repeat proteins | 0.555685 | 0.581018 | -0.24578 | 0.805852 | 0.935822 |
| O7b | Hydrogenase | 0.602939 | 0.579348 | 0.402218 | 0.687524 | 0.908513 |
| O8 | Transposon-related functions | 0.609425 | 0.578318 | 0.585472 | 0.55823 | 0.843041 |

**Table S5:** Excel table with genes that show strong median correlation with genes belonging to the different functional sub-categories of photosynthesis and respiration in Cyanobase, namely ATP synthase (H1), $CO_2$ fixation (H2), Cytochrome $b_6f$ complex (H3), NADH dehydrogenase (H5), PSI (H6), PSII (H7), PBS (H8) and Respiratory terminal oxidases (H10), but are not of the same functional sub-category. The thresholds for inclusion were set based on the median correlation of genes within these categories. For H2 and H3, whose members showed relatively low internal correlation, the threshold was set equal the 0.9 quantile, while a more relaxed threshold of 0.8 quantile was set for the other categories that showed higher internal correlation.

**[Supplementary Table S5 online]**

**Table S6:** Excel file including an overview of control and corresponding treatment conditions as described in the original publication of microarray experiments analyzed in the meta-analysis. Additionally, authors, title and PubMed reference of the studies are given in the table along with type of perturbation evoked in the corresponding experiments.

**[Supplementary Table S6 online]**

**Table S7:** Correlation between genes of the same functional main category and subcategory. **N**: Number of genes associated with the corresponding category in Cyanobase. **Mean**: Mean Spearman correlation between pairs of genes associated with each main category or subcategory. **Mean (rand)**: Mean Spearman correlation between random pairs of genes across the whole dataset. **Std (rand)**: Standard deviation of Mean (rand). **N Pairs**: Number of pairs within a main category. **P-value:** Significance derived using the one sample Wilcox rank test for deviation from 0. **FDR**: False discovery rates derived from the p-values using the Benjamini-Hochberg procedure.

| Cat. | Main-Function | N | Mean | Mean (rand) | Std (rand) | N Pairs | P-value | FDR |
|---|---|---|---|---|---|---|---|---|
| A | Amino acid biosynthesis | 97 | 0.037833 | 0.002863 | 0.002899 | 4656 | 1.77E-31 | 4.04E-31 |
| B | Biosynthesis of cofactors, prosthetic groups, and carriers | 125 | 0.01392 | 0.002779 | 0.002444 | 7626 | 1.87E-06 | 2.49E-06 |
| C | Cell envelope | 67 | 0.058169 | 0.002572 | 0.004449 | 2211 | 3.25E-30 | 6.51E-30 |
| D | Cellular processes | 80 | 0.018363 | 0.003271 | 0.003654 | 2775 | 0.00146 | 0.001797 |
| E | Central intermediary metabolism | 31 | 0.028493 | 0.001716 | 0.010003 | 465 | 0.090749 | 0.096799 |
| F | Energy metabolism | 93 | 0.023569 | 0.002379 | 0.003092 | 4278 | 1.28E-09 | 2.05E-09 |
| G | Fatty acid, phospholipid and sterol metabolism | 39 | 0.049742 | 0.002015 | 0.007801 | 741 | 2.14E-07 | 3.11E-07 |
| H | Photosynthesis and respiration | 143 | 0.135931 | 0.002653 | 0.002108 | 9870 | 0 | 0 |
| I | Purines, pyrimidines, nucleosides, and nucleotides | 43 | 0.097337 | 0.001283 | 0.007903 | 780 | 2.30E-33 | 6.15E-33 |
| J | Regulatory functions | 156 | 0.026341 | 0.002925 | 0.001877 | 10585 | 8.45E-50 | 3.38E-49 |
| K2 | DNA replication, restriction, modification, recombination, and repair | 75 | 0.046028 | 0.002721 | 0.004959 | 1770 | 1.92E-26 | 3.41E-26 |
| L | Transcription | 30 | 0.01903 | 0.002596 | 0.010603 | 435 | 0.294989 | 0.294989 |
| M | Translation | 168 | 0.129792 | 0.002829 | 0.001662 | 13861 | 0 | 0 |
| N | Transport and binding proteins | 200 | 0.017506 | 0.002839 | 0.001465 | 19110 | 1.19E-33 | 3.82E-33 |
| O | Other categories | 369 | 0.005903 | 0.002547 | 0.001516 | 23005 | 0.004505 | 0.005149 |
| | | | | | | | | |
| **SCat.** | **Sub-Function** | **N** | **Mean** | **Mean (rand)** | **Std (rand)** | **N Pairs** | **P-value** | **FDR** |
| A1 | Aromatic amino acid family | 28 | 0.033699 | 0.004734 | 0.011443 | 378 | 0.000405 | 0.001081 |
| A2 | Aspartate family | 12 | 0.032788 | 0.003106 | 0.025527 | 66 | 0.303724 | 0.422572 |
| A3 | Branched chain family | 13 | 0.058673 | 0.006421 | 0.023034 | 78 | 0.015923 | 0.031845 |
| A4 | Glutamate family / Nitrogen assimilation | 35 | 0.032142 | 0.00177 | 0.007815 | 595 | 0.001351 | 0.003203 |
| A6 | Serine family / Sulfur assimilation | 9 | 0.143821 | 0.003673 | 0.032428 | 36 | 0.000585 | 0.001496 |
| B1 | Biotin | 3 | -0.03797 | 0.019608 | 0.11408 | 3 | NA | NA |
| B2 | Folic acid | 5 | 0.03557 | 0.009659 | 0.0635 | 10 | 0.492188 | 0.605769 |
| B3 | Cobalamin, heme, phycobilin and porphyrin | 48 | 0.027662 | 0.002915 | 0.005649 | 1081 | 0.012075 | 0.024929 |
| B4 | Lipoate | 3 | 0.01421 | 0.0047 | 0.10858 | 3 | NA | NA |
| B5 | Menaquinone and ubiquinone | 9 | 0.060613 | 0.002132 | 0.033635 | 36 | 0.126644 | 0.207825 |

| | | | | | | | | |
|---|---|---|---|---|---|---|---|---|
| B6 | Molybdopterin | 6 | 0.289474 | -0.00158 | 0.056503 | 15 | 0.000854 | 0.002103 |
| B7 | Pantothenate | 4 | 0.18146 | 0.008999 | 0.077225 | 6 | 0.15625 | 0.243902 |
| B8 | Pyridoxine | 3 | 0.166208 | 0.013771 | 0.117264 | 3 | NA | NA |
| B9 | Riboflavin | 5 | 0.04939 | -0.00133 | 0.064619 | 10 | 0.375 | 0.5 |
| B10 | Thioredoxin, glutaredoxin, and glutathione | 18 | 0.000192 | 0.001842 | 0.018133 | 153 | 0.894217 | 0.923063 |
| B12 | Thiamin | 4 | 0.036144 | 0.002481 | 0.090335 | 6 | 0.4375 | 0.571429 |
| B13 | Carotenoid | 9 | 0.055042 | 0.007289 | 0.03711 | 36 | 0.271629 | 0.39375 |
| B15 | Quinolinate | 2 | -0.14428 | 0.016982 | 0.164196 | 1 | NA | NA |
| B17 | Others | 2 | -0.22286 | 0.053807 | 0.21342 | 1 | NA | NA |
| B18 | Nicotinate and nicotinamide | 4 | 0.02012 | -0.00267 | 0.095846 | 6 | 0.6875 | 0.758621 |
| C1 | Membranes, lipoproteins, and porins | 12 | 0.123374 | -0.00352 | 0.024039 | 66 | 0.000271 | 0.000755 |
| C2 | Murein sacculus and peptidoglycan | 24 | 0.114196 | 0.001225 | 0.010738 | 276 | 1.14E-14 | 6.60E-14 |
| C3 | Surface polysaccharides, lipopolysaccharides and antigens | 28 | 0.037842 | 0.002555 | 0.009675 | 378 | 0.002833 | 0.006252 |
| C4 | Surface structures | 3 | -0.0643 | -0.00747 | 0.107911 | 3 | NA | NA |
| D1 | Cell division | 14 | 0.040457 | 0.004366 | 0.021474 | 91 | 0.239005 | 0.360168 |
| D2 | Cell killing | 4 | -0.11403 | 0.005627 | 0.1015 | 3 | NA | NA |
| D3 | Chaperones | 16 | 0.149513 | -0.001 | 0.019445 | 120 | 3.16E-08 | 1.06E-07 |
| D4 | Detoxification | 8 | -0.03366 | 0.006014 | 0.059447 | 10 | 0.492188 | 0.605769 |
| D5 | Protein and peptide secretion | 15 | 0.027398 | 0.002238 | 0.019674 | 105 | 0.241988 | 0.360168 |
| D6 | Transformation | 7 | 0.035229 | -0.00206 | 0.056788 | 15 | 0.276855 | 0.39375 |
| D7 | Chemotaxis | 16 | 0.16441 | 0.004529 | 0.017599 | 120 | 7.82E-11 | 3.58E-10 |
| E1 | Amino sugars | 1 | NA | NA | NA | 0 | NA | NA |
| E3 | Other | 7 | 0.034647 | 0.000154 | 0.046592 | 21 | 0.320457 | 0.436368 |
| E4 | Phosphorus compounds | 3 | 0.272135 | 0.002134 | 0.125029 | 3 | NA | NA |
| E6 | Polysaccharides and glycoproteins | 20 | 0.017977 | 0.007266 | 0.014449 | 190 | 0.550623 | 0.6438 |
| F2 | Amino acids and amines | 25 | 0.01777 | 0.00445 | 0.010899 | 300 | 0.098358 | 0.165655 |
| F9 | Glycolysis | 15 | 0.093482 | 0.001457 | 0.019304 | 105 | 0.002103 | 0.004806 |
| F10 | Pentose phosphate pathway | 8 | 0.187069 | 0.001124 | 0.0433 | 28 | 0.004778 | 0.010193 |
| F11 | Pyruvate dehydrogenase | 4 | 0.533959 | 0.000472 | 0.079645 | 6 | 0.03125 | 0.057143 |
| F12 | Sugars | 20 | -0.00935 | 0.003613 | 0.015568 | 190 | 0.553266 | 0.6438 |
| F13 | TCA cycle | 9 | 0.001339 | 0.002922 | 0.035624 | 36 | 0.969058 | 0.969058 |
| F14 | Pyruvate and acetyl-CoA metabolism | 8 | -0.01069 | -0.00136 | 0.041631 | 28 | 0.955322 | 0.969058 |
| F15 | Glycolate pathway | 4 | -0.02768 | -0.01196 | 0.085137 | 6 | 0.84375 | 0.9 |
| G | Fatty acid, phospholipid and sterol metabolism | 39 | 0.049742 | 0.001724 | 0.006826 | 741 | 2.14E-07 | 6.84E-07 |
| H1 | ATP synthase | 10 | 0.728877 | 0.006634 | 0.033746 | 36 | 2.91E-11 | 1.43E-10 |
| H10 | Respiratory terminal oxidases | 9 | 0.311802 | -0.00165 | 0.029341 | 36 | 2.04E-10 | 8.69E-10 |

| | | | | | | | | |
|---|---|---|---|---|---|---|---|---|
| H2 | CO2 fixation | 15 | 0.189946 | 0.000152 | 0.020611 | 105 | 6.93E-09 | 2.46E-08 |
| H3 | Cytochrome b6/f complex | 9 | 0.154524 | 0.008033 | 0.035434 | 36 | 0.023891 | 0.044971 |
| H5 | NADH dehydrogenase | 23 | 0.209064 | 0.00261 | 0.012502 | 231 | 1.24E-22 | 9.53E-22 |
| H6 | Photosystem I | 16 | 0.380604 | -0.00151 | 0.019433 | 120 | 1.60E-18 | 1.02E-17 |
| H7 | Photosystem II | 28 | 0.263499 | 0.001603 | 0.011833 | 378 | 2.83E-45 | 4.52E-44 |
| H8 | Phycobilisome | 18 | 0.292606 | 0.002204 | 0.017503 | 153 | 4.12E-10 | 1.65E-09 |
| H9 | Soluble electron carriers | 15 | -0.00336 | -0.00032 | 0.021993 | 105 | 0.867973 | 0.91066 |
| I2 | Interconversions and salvage of nucleosides and nucleotides | 7 | 0.069975 | -0.00317 | 0.084341 | 6 | 0.15625 | 0.243902 |
| I3 | Purine ribonucleotide biosynthesis | 24 | 0.087778 | 0.002273 | 0.012958 | 276 | 6.34E-12 | 3.38E-11 |
| I4 | Pyrimidine ribonucleotide biosynthesis | 12 | 0.119045 | 0.002531 | 0.027699 | 66 | 0.00014 | 0.000409 |
| J | Regulatory functions | 156 | 0.026341 | 0.002609 | 0.00202 | 10585 | 8.45E-50 | 1.80E-48 |
| K2 | DNA replication, restriction, modification, recombination, and repair | 75 | 0.046028 | 0.002943 | 0.004521 | 1770 | 1.92E-26 | 1.76E-25 |
| L1 | Degradation of RNA | 7 | 0.136861 | -0.00131 | 0.04701 | 21 | 0.021571 | 0.041835 |
| L2 | RNA synthesis, modification, and DNA transcription | 23 | -0.00481 | 0.00268 | 0.015728 | 253 | 0.52535 | 0.634385 |
| M1 | Aminoacyl tRNA synthetases and tRNA modification | 43 | 0.110762 | 0.003204 | 0.006225 | 903 | 5.93E-43 | 7.58E-42 |
| M2 | Degradation of proteins, peptides, and glycopeptides | 26 | 0.061574 | 0.001555 | 0.012349 | 325 | 2.76E-06 | 8.42E-06 |
| M3 | Nucleoproteins | 7 | -0.02735 | 0.00164 | 0.043121 | 21 | 0.657827 | 0.751803 |
| M4 | Protein modification and translation factors | 29 | 0.073099 | 0.003193 | 0.011408 | 406 | 2.66E-09 | 1.00E-08 |
| M5 | Ribosomal proteins: synthesis and modification | 63 | 0.415678 | 0.002573 | 0.005338 | 1891 | 6.40E-277 | 4.10E-275 |
| N | Transport and binding proteins | 200 | 0.017506 | 0.00258 | 0.001504 | 19110 | 1.19E-33 | 1.27E-32 |
| O1 | Adaptations and atypical conditions | 12 | -0.01237 | 0.003394 | 0.02515 | 66 | 0.682659 | 0.758621 |
| O3 | Drug and analog sensitivity | 23 | 0.006561 | 0.004437 | 0.011642 | 253 | 0.720419 | 0.781471 |
| O7 | Other | 178 | 0.005186 | 0.002661 | 0.001819 | 10153 | 0.049072 | 0.084882 |
| O7a | WD repeat proteins | 6 | 0.181198 | -0.00109 | 0.063964 | 10 | 0.037109 | 0.065972 |
| O7b | Hydrogenase | 15 | 0.039815 | 0.002259 | 0.019748 | 105 | 0.489873 | 0.605769 |
| O8 | Transposon-related functions | 135 | 0.428559 | 0.005893 | 0.016465 | 136 | 1.34E-22 | 9.53E-22 |

## *6. Supplemental references*


1 Hihara, Y., Kamei, A., Kanehisa, M., Kaplan, A. & Ikeuchi, M. DNA microarray analysis of cyanobacterial gene expression during acclimation to high light. *Plant Cell* **13**, 793-806 (2001).

2 Postier, B. L. *et al.* The construction and use of bacterial DNA microarrays based on an optimized two-stage PCR strategy. *BMC Genomics* **4**, 23 (2003).

3 Tu, C. J., Shrager, J., Burnap, R. L., Postier, B. L. & Grossman, A. R. Consequences of a deletion in dspA on transcript accumulation in Synechocystis sp. strain PCC6803. *J. Bacteriol.* **186**, 3889-3902 (2004).

4 Zhang, Z., Pendse, N. D., Phillips, K. N., Cotner, J. B. & Khodursky, A. Gene expression patterns of sulfur starvation in Synechocystis sp. PCC 6803. *BMC Genomics* **9**, 344 (2008).

5 Singh, A. K. *et al.* Integration of carbon and nitrogen metabolism with energy production is crucial to light acclimation in the cyanobacterium Synechocystis. *Plant Physiol.* **148**, 467-478 (2008).

6 Georg, J. *et al.* Evidence for a major role of antisense RNAs in cyanobacterial gene regulation. *Mol. Syst. Biol.* **5**, 305 (2009).

7 Nakamura, Y., Kaneko, T., Hirosawa, M., Miyajima, N. & Tabata, S. CyanoBase, a www database containing the complete nucleotide sequence of the genome of Synechocystis sp. strain PCC6803. *Nucleic Acids Res.* **26**, 63-67 (1998).

8 Aguirre von Wobeser, E. *et al.* Concerted Changes in Gene Expression and Cell Physiology of the Cyanobacterium Synechocystis sp. Strain PCC 6803 during Transitions between Nitrogen and Light-Limited Growth. *Plant Physiol.* **155**, 1445-1457 (2011).

9 Dickson, D. J., Luterra, M. D. & Ely, R. L. Transcriptomic responses of Synechocystis sp. PCC 6803 encapsulated in silica gel. *Appl. Microbiol. Biotechnol.* **96**, 183-196 (2012).

10 Rowland, J. G. *et al.* Identification of components associated with thermal acclimation of photosystem II in Synechocystis sp. PCC6803. *PLoS One* **5**, e10511 (2010).

11 Panichkin, V. B. *et al.* Serine/Threonine Protein Kinase SpkA in Synechocystis sp. Strain PCC 6803 Is a Regulator of Expression of Three Putative pilA Operons, Formation of Thick Pili, and Cell Motility. *J. Bacteriol.* **188**, 7696-7699 (2006).

12 Shoumskaya, M. A. *et al.* Identical Hik-Rre Systems Are Involved in Perception and Transduction of Salt Signals and Hyperosmotic Signals but Regulate the Expression of Individual Genes to Different Extents in Synechocystis. *J. Biol. Chem.* **280**, 21531-21538 (2005).

13 Prakash, J. S. S. *et al.* DNA supercoiling regulates the stress-inducible expression of genes in the cyanobacterium Synechocystis. *Molecular BioSystems* **5**, 1904-1912 (2009).
